# Correction: Liu et al. Identification of C_21_ Steroidal Glycosides from *Gymnema sylvestre* (Retz.) and Evaluation of Their Glucose Uptake Activities. *Molecules* 2021, *26*, 6549

**DOI:** 10.3390/molecules27175718

**Published:** 2022-09-05

**Authors:** Meiyu Liu, Tongxi Zhou, Jinyan Zhang, Guangfeng Liao, Rumei Lu, Xinzhou Yang

**Affiliations:** 1 School of Pharmaceutical Sciences, Guangxi University of Chinese Medicine, Nanning 530200, China; 2 School of Pharmaceutical Sciences, South-Central University for Nationalities, Wuhan 430074, China

After careful examination, we found that the structures [1] of compounds **1**–**4** in Figure 1 were not correct due to oversight. The oxygen atoms in the acyl groups of compounds **1**–**4** were missing. The authors would like to apologize for any inconvenience caused to readers by this correction. Replacing this figure will not affect the results or conclusions of the paper. The manuscript will be updated and the original will remain online on the article webpage, with reference to this correction.

## Figures and Tables

**Figure 1:**
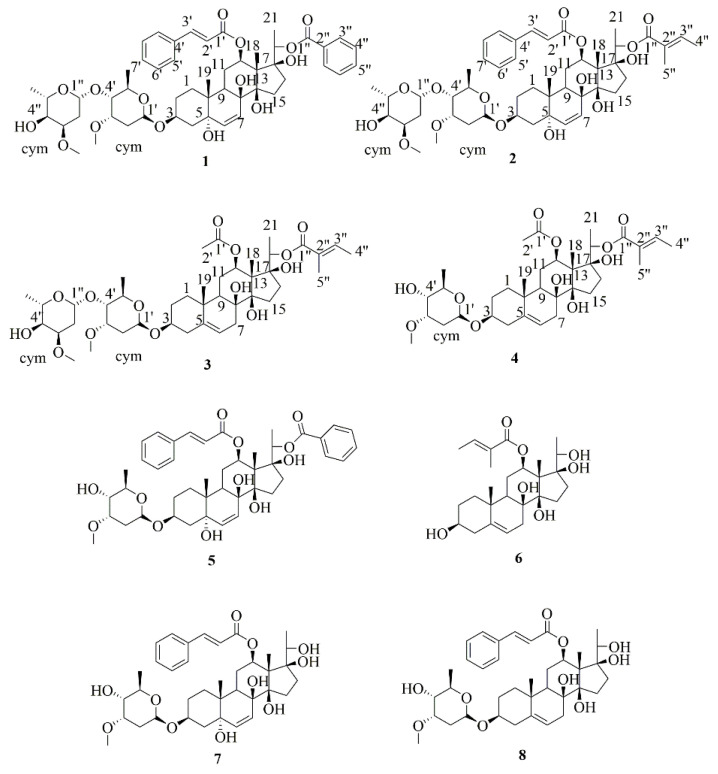
The structures of compounds **1**–**8**.
