# Supplementary material for: Identification of C21 Steroidal Glycosides from Gymnema sylvestre (Retz.) and Evaluation of Their Glucose Uptake Activities
Source: Molecules. 2021 Oct 29;26(21):6549. doi: 10.3390/molecules26216549 (PMC8588469; doi:10.3390/molecules26216549)
Supplement: Supplementary file 1 [file molecules-26-06549-s001.zip › molecules-1378917-supplementary.pdf]

Supporting Information

Identification of C_{21} steroidal glycosides from *Gymnema sylvestre* (Retz.) and evaluation their glucose uptake activities

Meiyu Liu [1], Tongxi Zhou [2], Jinyan Zhang [1], Guangfeng Liao [1], Rumei Lu [1,*] and Xinzhou Yang [2,*]

[1] *School of Pharmaceutical Sciences, Guangxi University of Chinese medicine, Nanning, 530200, China*

[2] *School of Pharmaceutical Sciences, South-Central University for Nationalities, Wuhan 430074, China;*

*Corresponding authors E-mail address: lurm@gxtcmu.edu.cn (R.M. Lu); xzyang@mail.scuec.edu.cn (X.Z. Yang).

CONTENT

**Figure S1** HRESIMS spectrum of **1**
**Figure S2** ^{1}H NMR spectrum (600 MHz, $CDCl_3$) of **1**
**Figure S3** ^{13}C NMR spectrum (150 MHz, $CDCl_3$) of **1**
**Figure S4** DEPT 135° spectrum (150 MHz, $CDCl_3$) of **1**
**Figure S5** HSQC spectrum of **1**
**Figure S6** COSY spectrum of **1**
**Figure S7** HMBC spectrum of **1**
**Figure S8** NOESY spectrum of **1**
**Figure S9** 1D TOCSY spectrum of **1**
**Figure S10** 2D TOCSY spectrum of **1**
**Figure S11** UV spectrum of **1**
**Figure S12** IR spectrum of **1**
**Figure S13** HRESIMS spectrum of **2**
**Figure S14** ^{1}H NMR spectrum (600 MHz, $CDCl_3$) of **2**
**Figure S15** ^{13}C NMR spectrum (150 MHz, $CDCl_3$) of **2**
**Figure S16** DEPT 135° spectrum (150 MHz, $CDCl_3$) of **2**
**Figure S1**7 HSQC spectrum of **2**
**Figure S18** COSY spectrum of **2**
**Figure S19** HMBC spectrum of **2**
**Figure S20** NOESY spectrum of **2**
**Figure S21** 1D TOCSY spectrum of **2**
**Figure S22** 2D TOCSY spectrum of **2**
**Figure S23** UV spectrum of **2**
**Figure S24** IR spectrum of **2**
**Figure S25** HRESIMS spectrum of **3**
**Figure S26** ^{1}H NMR spectrum (600 MHz, $CDCl_3$) of **3**
**Figure S27** ^{13}C NMR spectrum (150 MHz, $CDCl_3$) of **3**
**Figure S28** DEPT 135° spectrum (150 MHz, $CDCl_3$) of **3**

**Figure S29** HSQC spectrum of **3**
**Figure S30** COSY spectrum of **3**
**Figure S31** HMBC spectrum of **3**
**Figure S32** NOESY spectrum of **3**
**Figure S33** 1D TOCSY spectrum of **3**
**Figure S34** 2D TOCSY spectrum of **3**
**Figure S35** UV spectrum of **3**
**Figure S36** IR spectrum of **3**
**Figure S37** HRESIMS spectrum of **4**
**Figure S38** ^{1}H NMR spectrum (600 MHz, $CDCl_3$) of **4**
**Figure S39** ^{13}C NMR spectrum (150 MHz, $CDCl_3$) of **4**
**Figure S40** DEPT 135° spectrum (150 MHz, $CDCl_3$) of **4**
**Figure S41** HSQC spectrum of **4**
**Figure S42** COSY spectrum of **4**
**Figure S43** HMBC spectrum of **4**
**Figure S44** NOESY spectrum of **4**
**Figure S45** 1D TOCSY spectrum of **4**
**Figure S46** 2D TOCSY spectrum of **4**
**Figure S47** UV spectrum of **4**
**Figure S48** IR spectrum of **4**

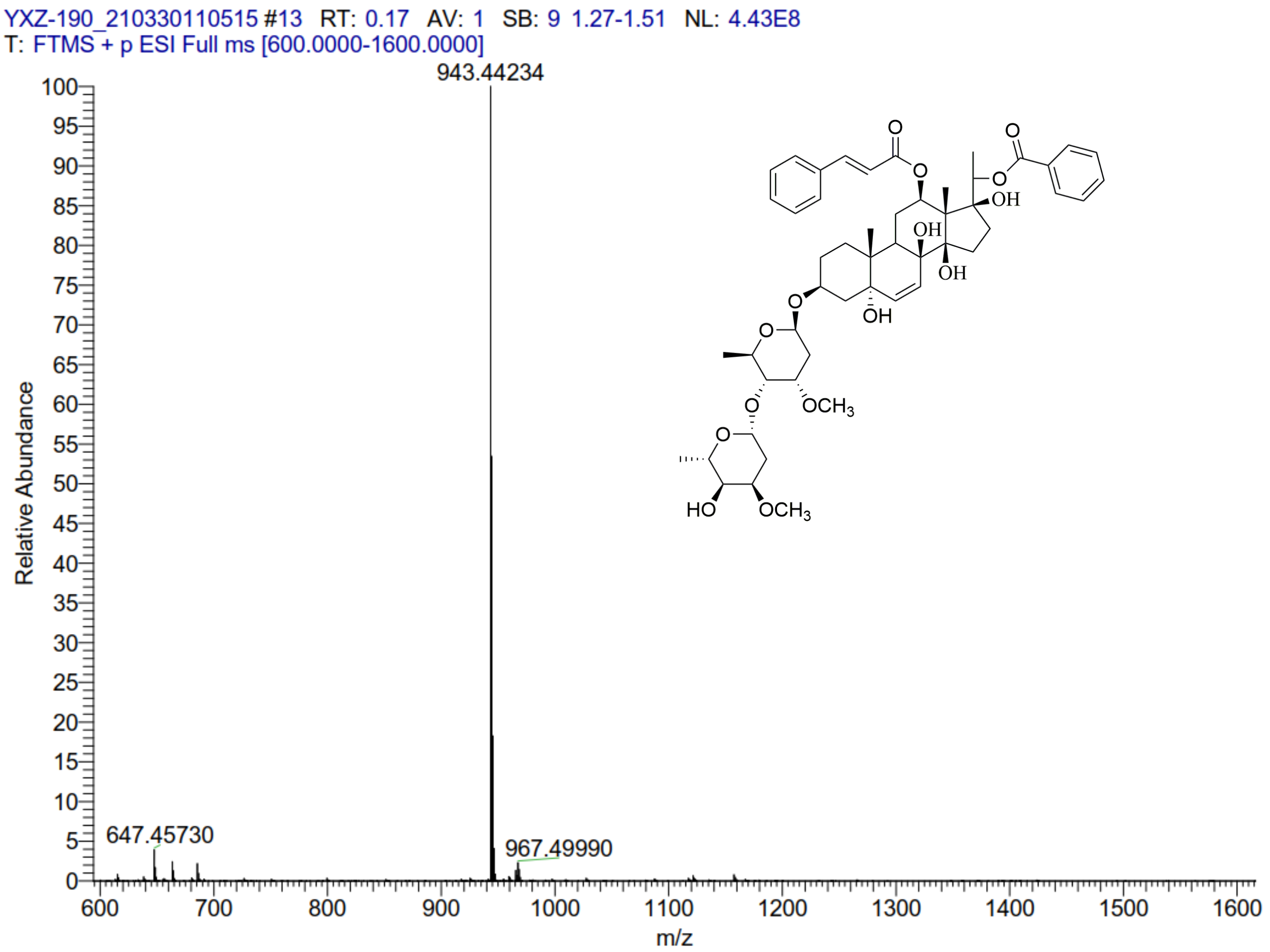

Figure S1 HRESIMS spectrum of **1**

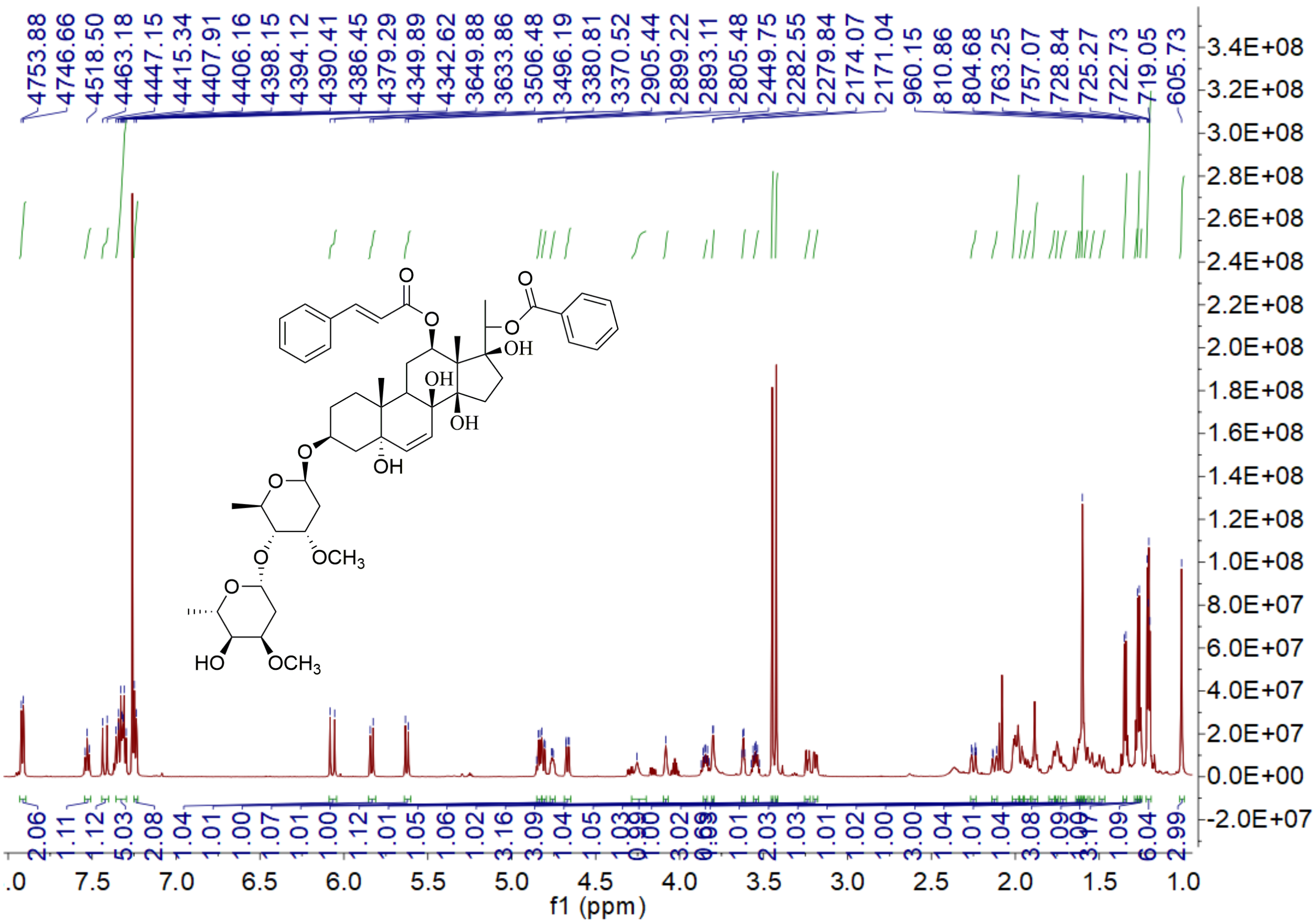

Figure S2 ^{1}H NMR spectrum (600 MHz, $CDCl_3$) of **1**

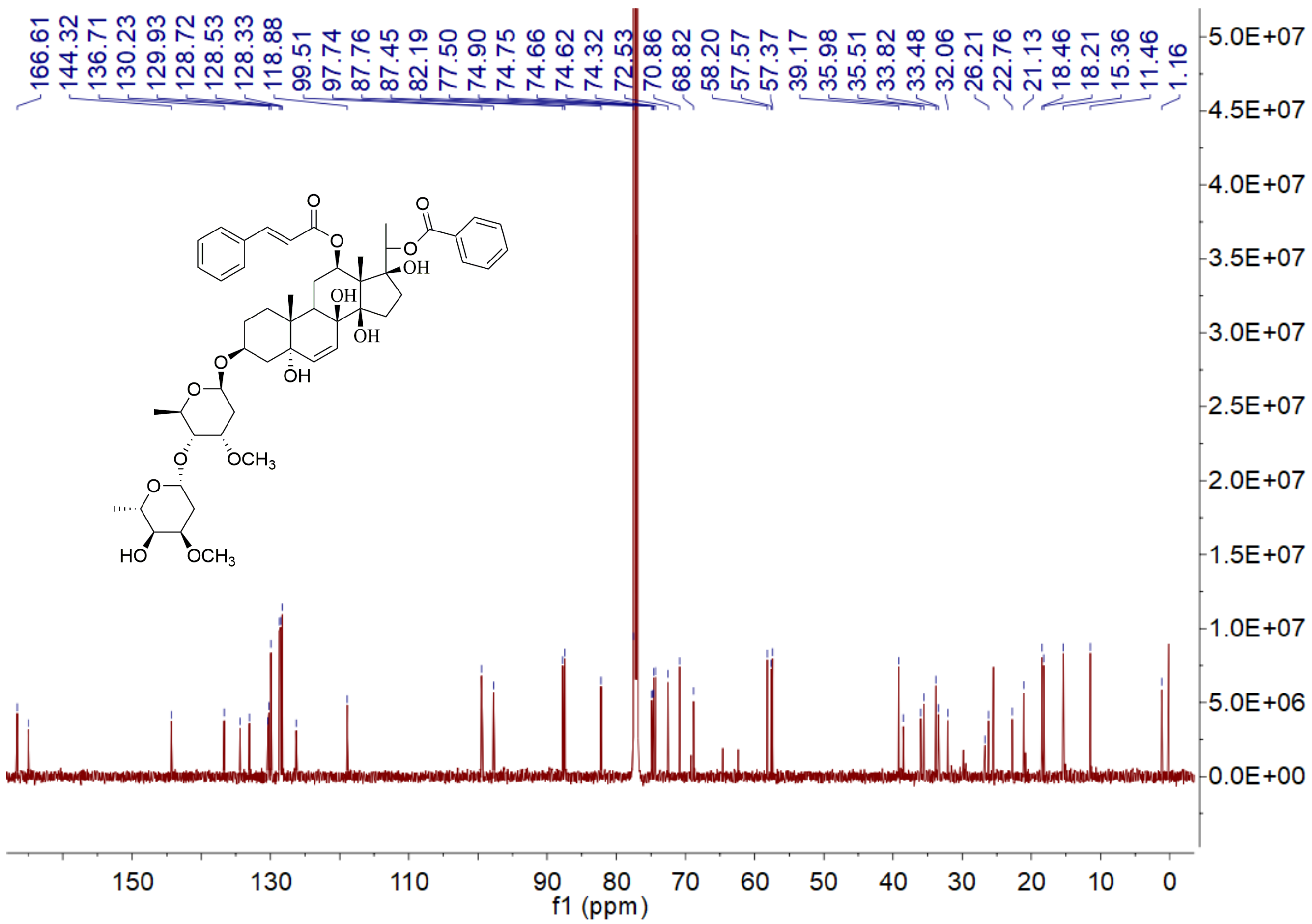

Figure S3 ^{13}C NMR spectrum (150 MHz, $CDCl_3$) of **1**

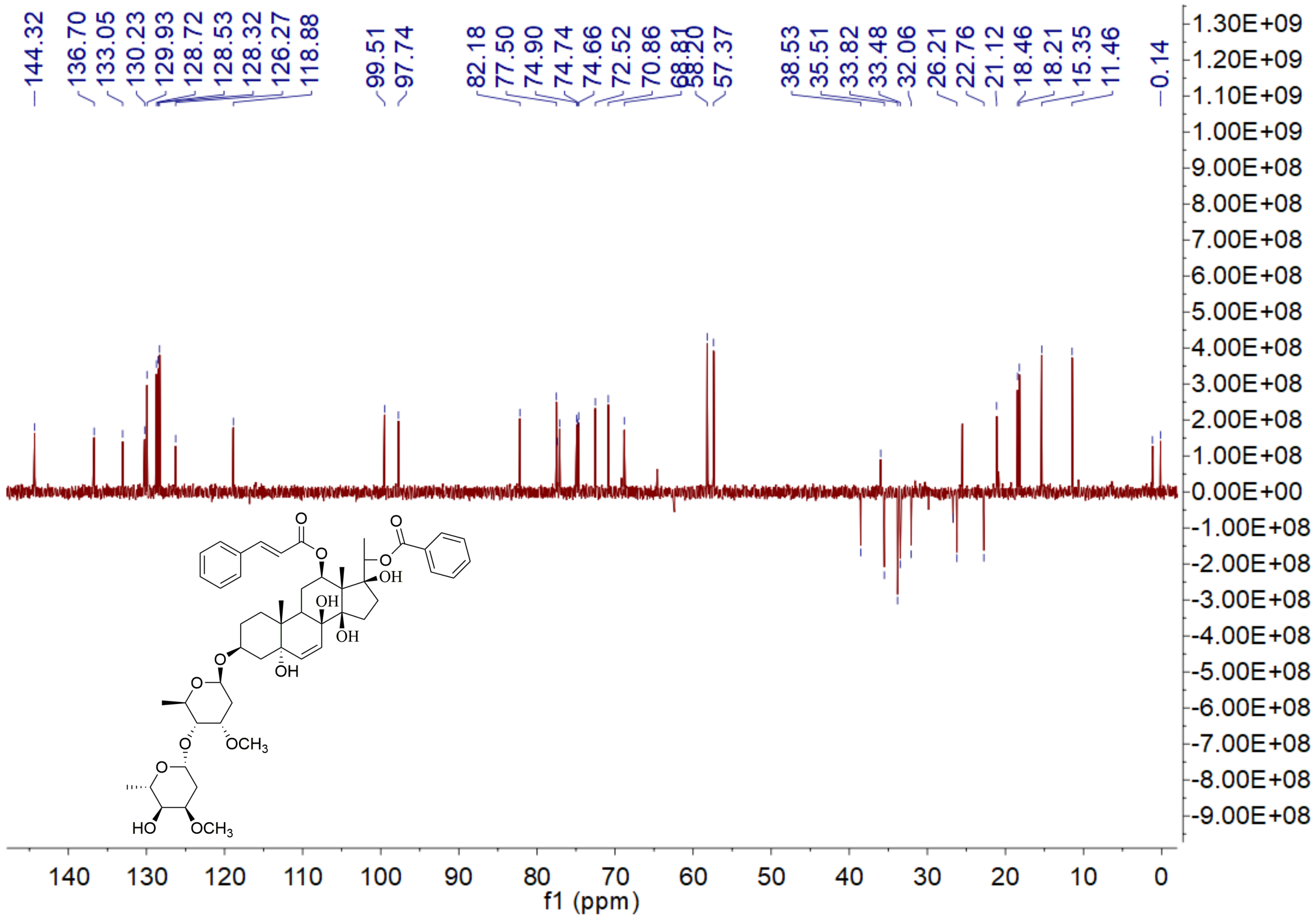

Figure S4 DEPT 135° spectrum (150 MHz, $CDCl_3$) of **1**

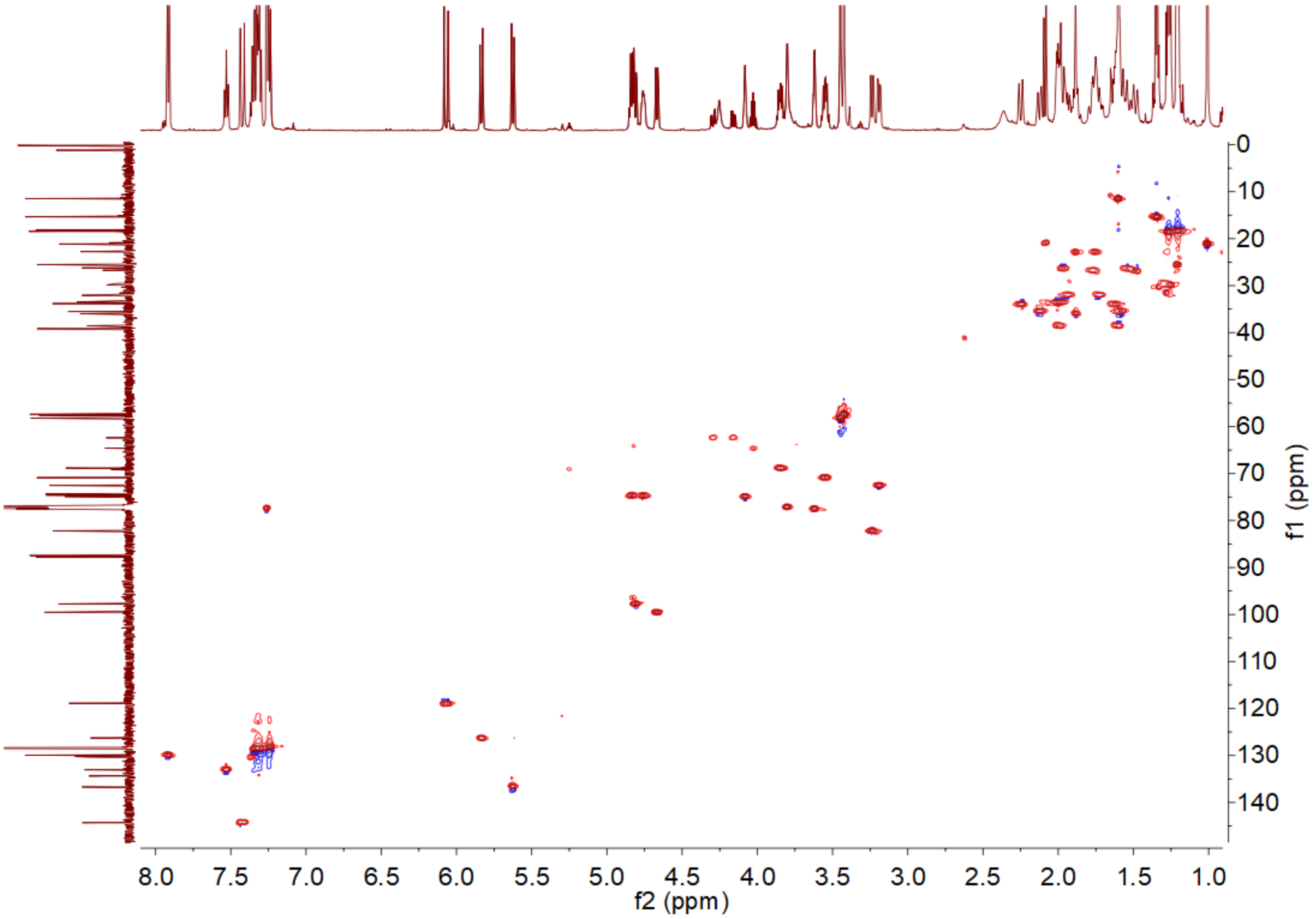

Figure S5 HSQC spectrum of **1**

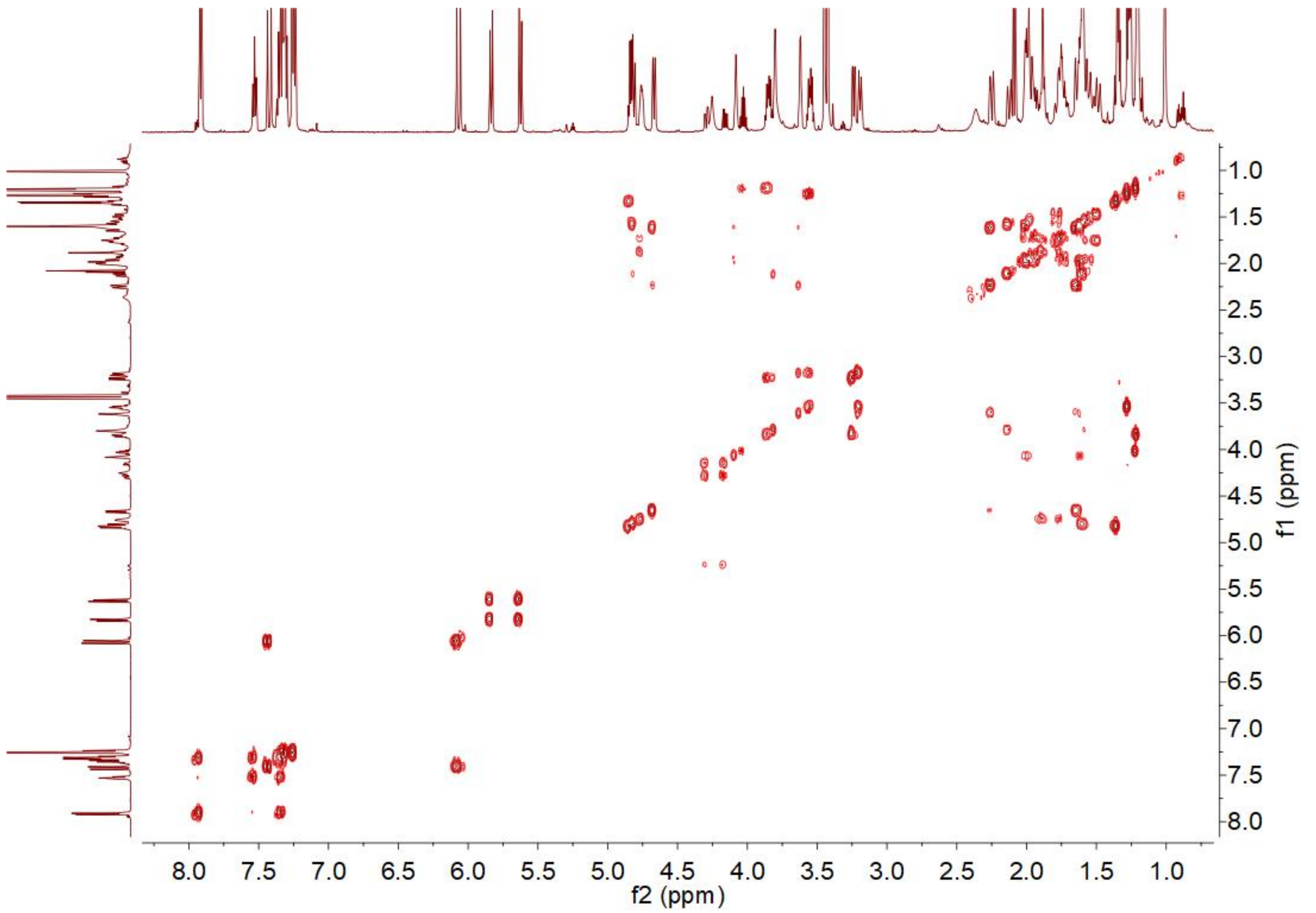

Figure S6 COSY spectrum of **1**

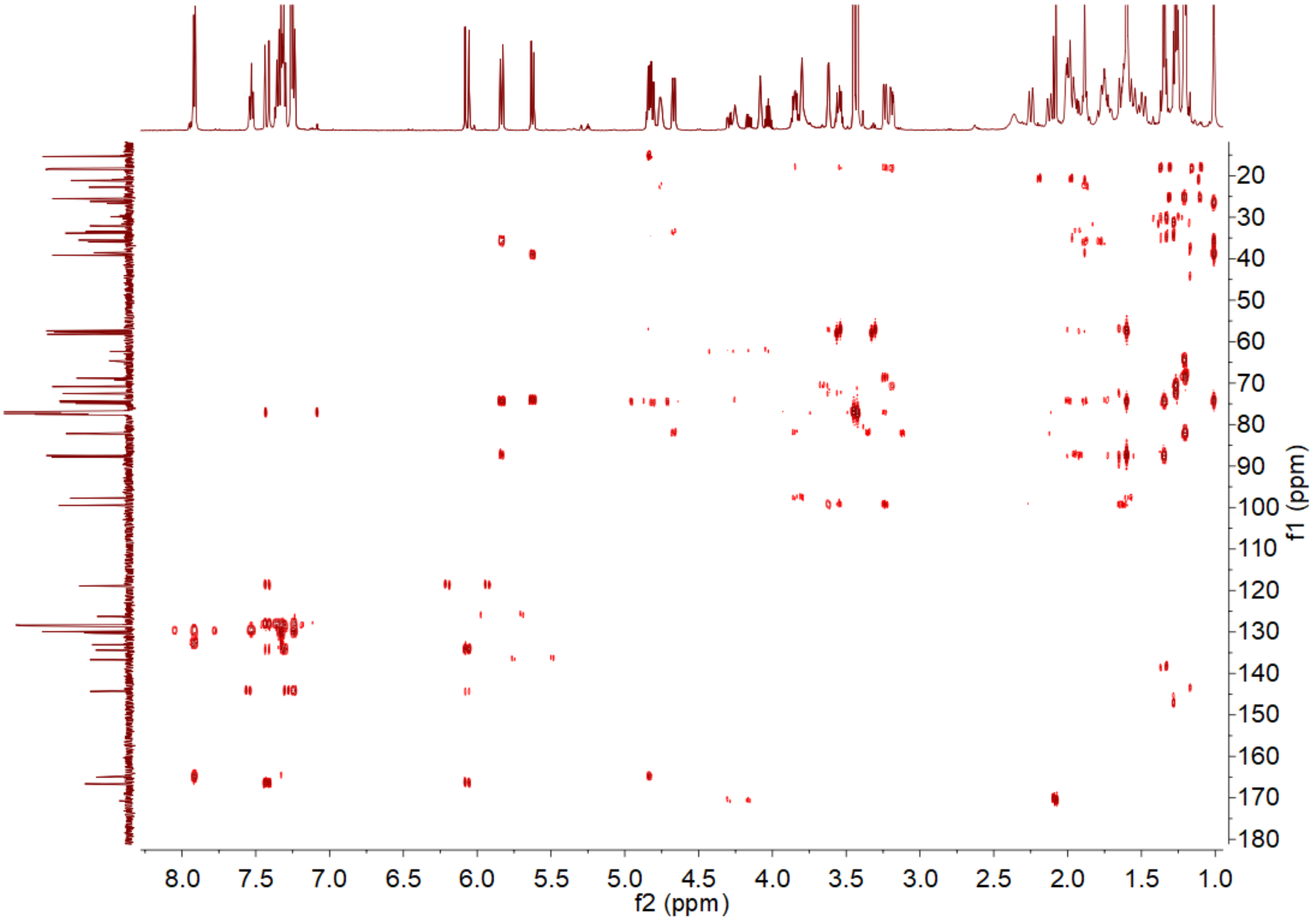

Figure S7 HMBC spectrum of **1**

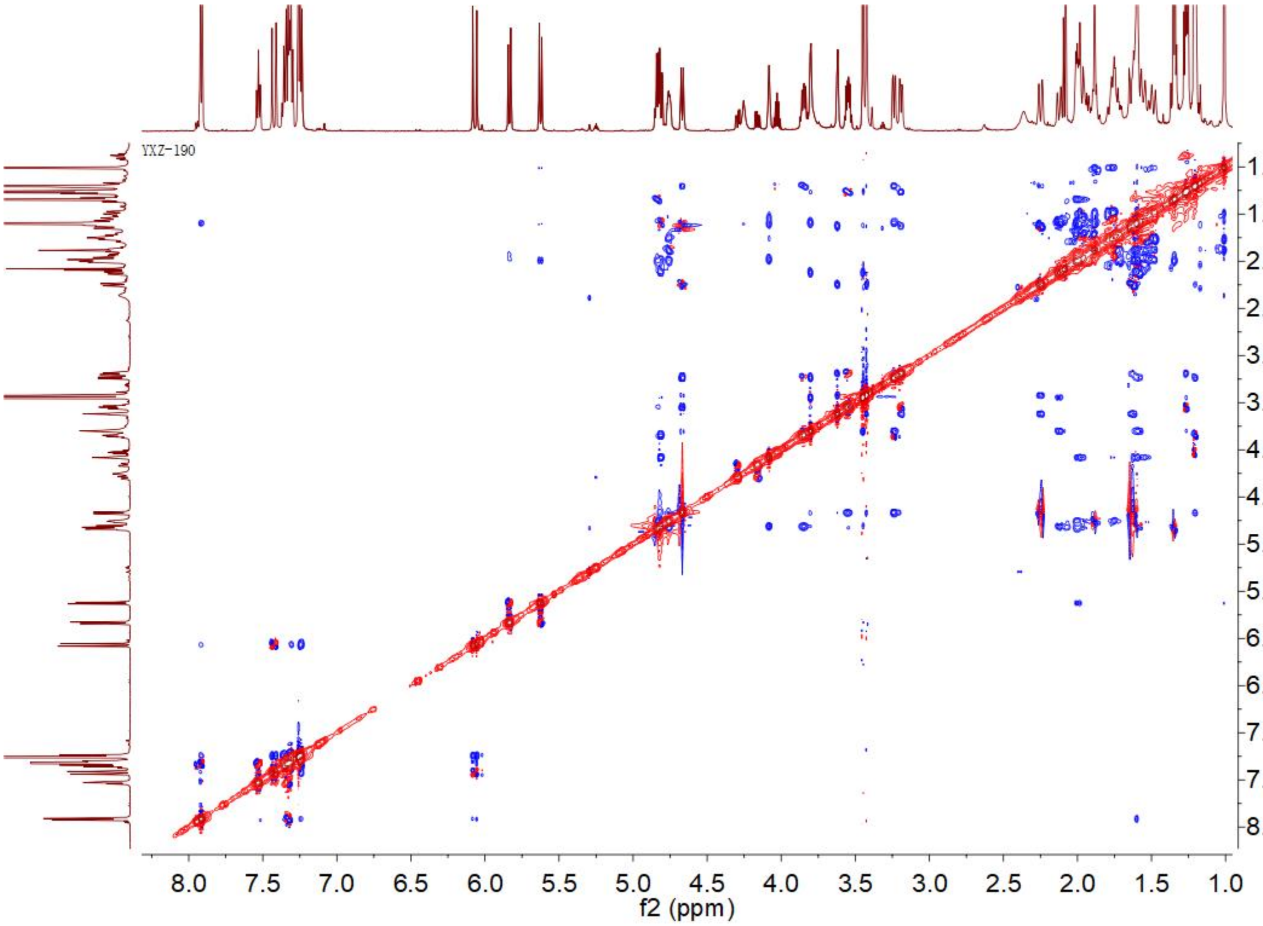

Figure S8 NOESY spectrum of **1**

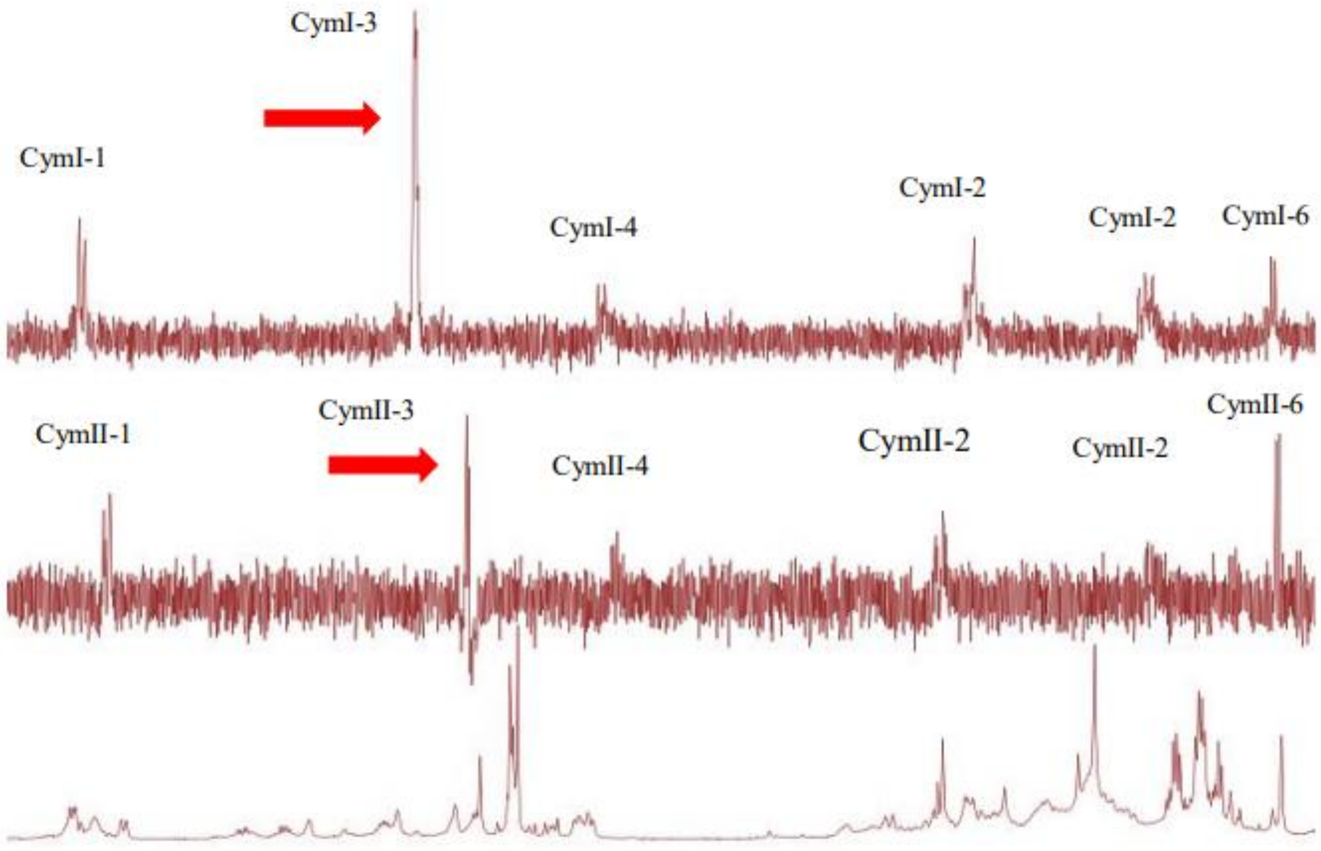

Figure S9 1D TOCSY spectrum of **1**

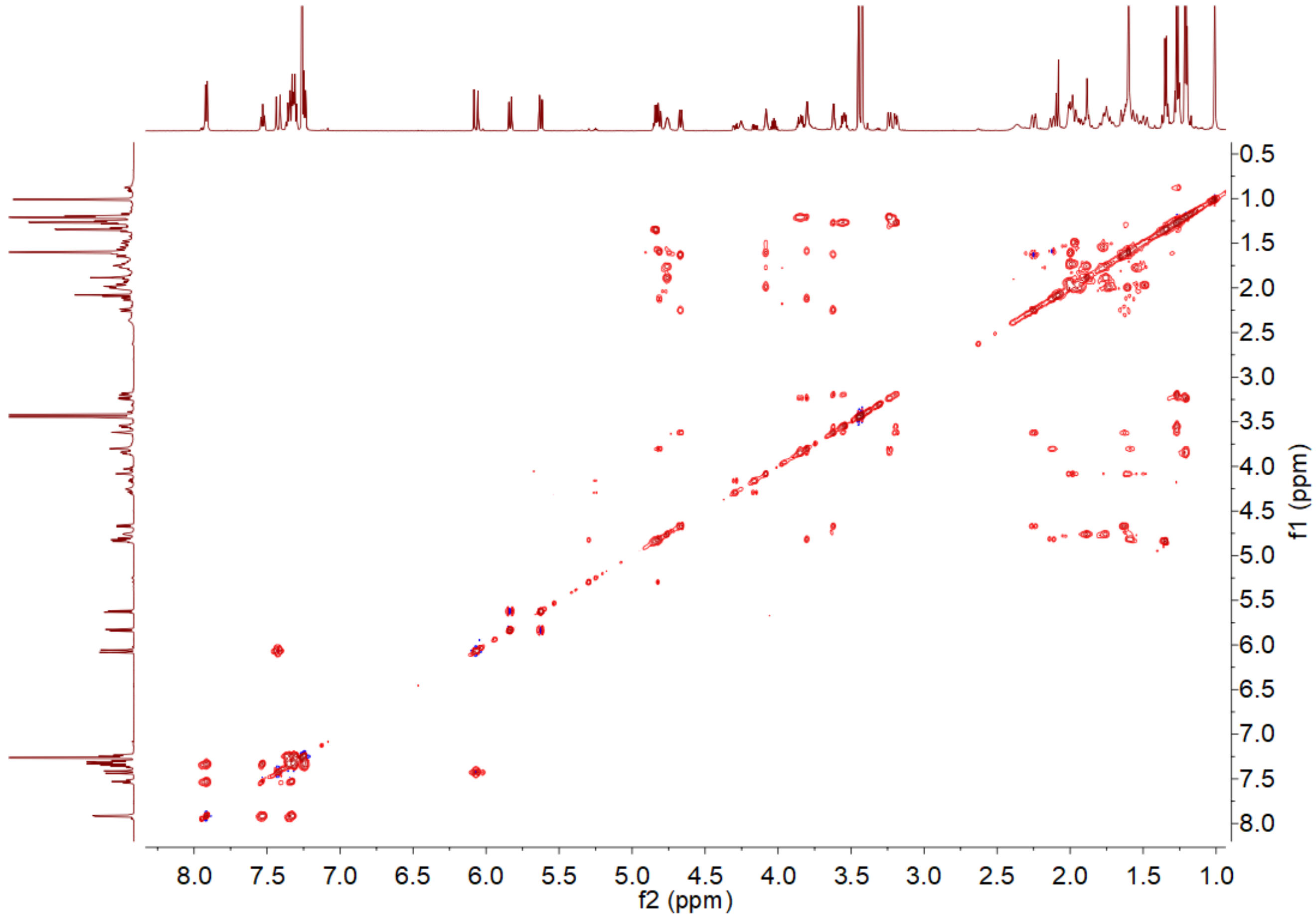

Figure S10 2D TOCSY spectrum of **1**

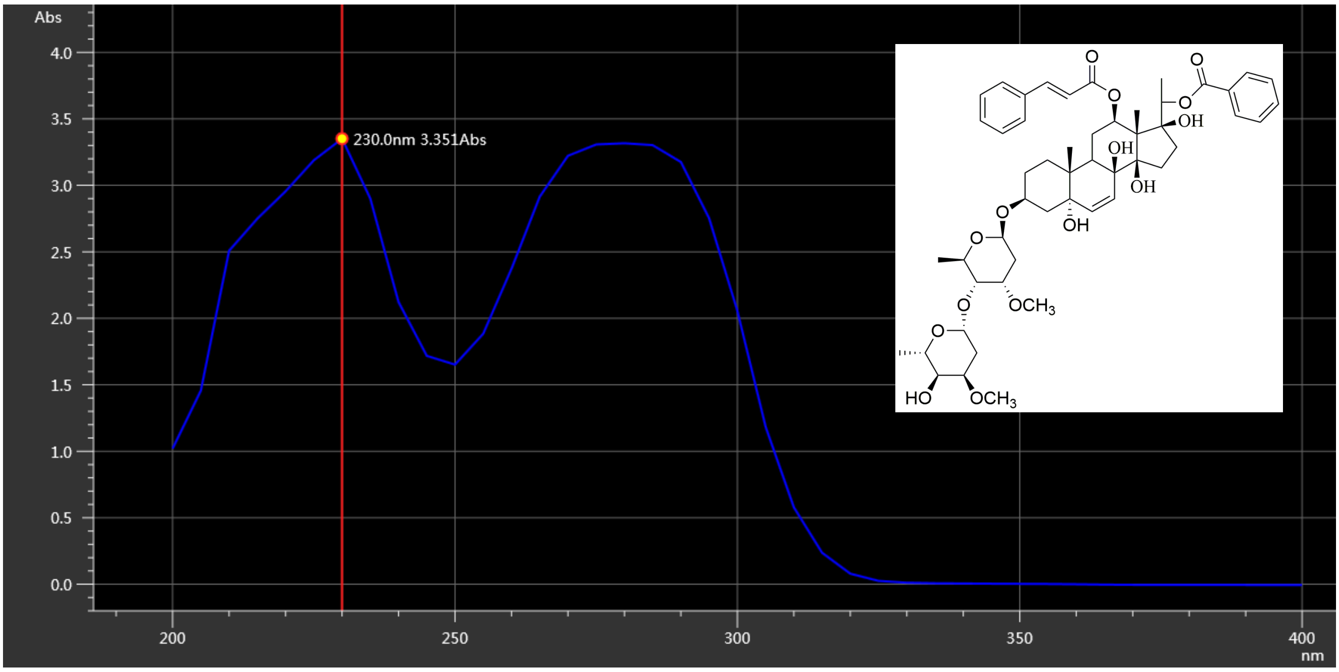

Figure S11 UV spectrum of **1**

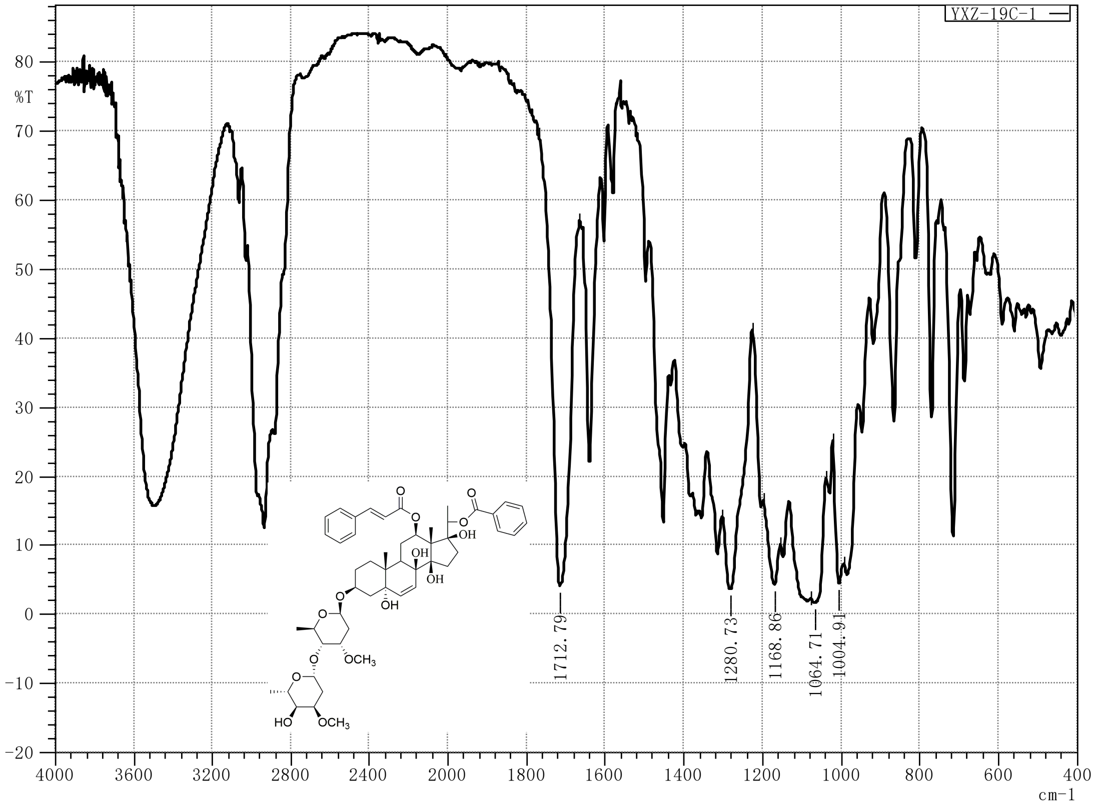

Figure S12 IR spectrum of **1**

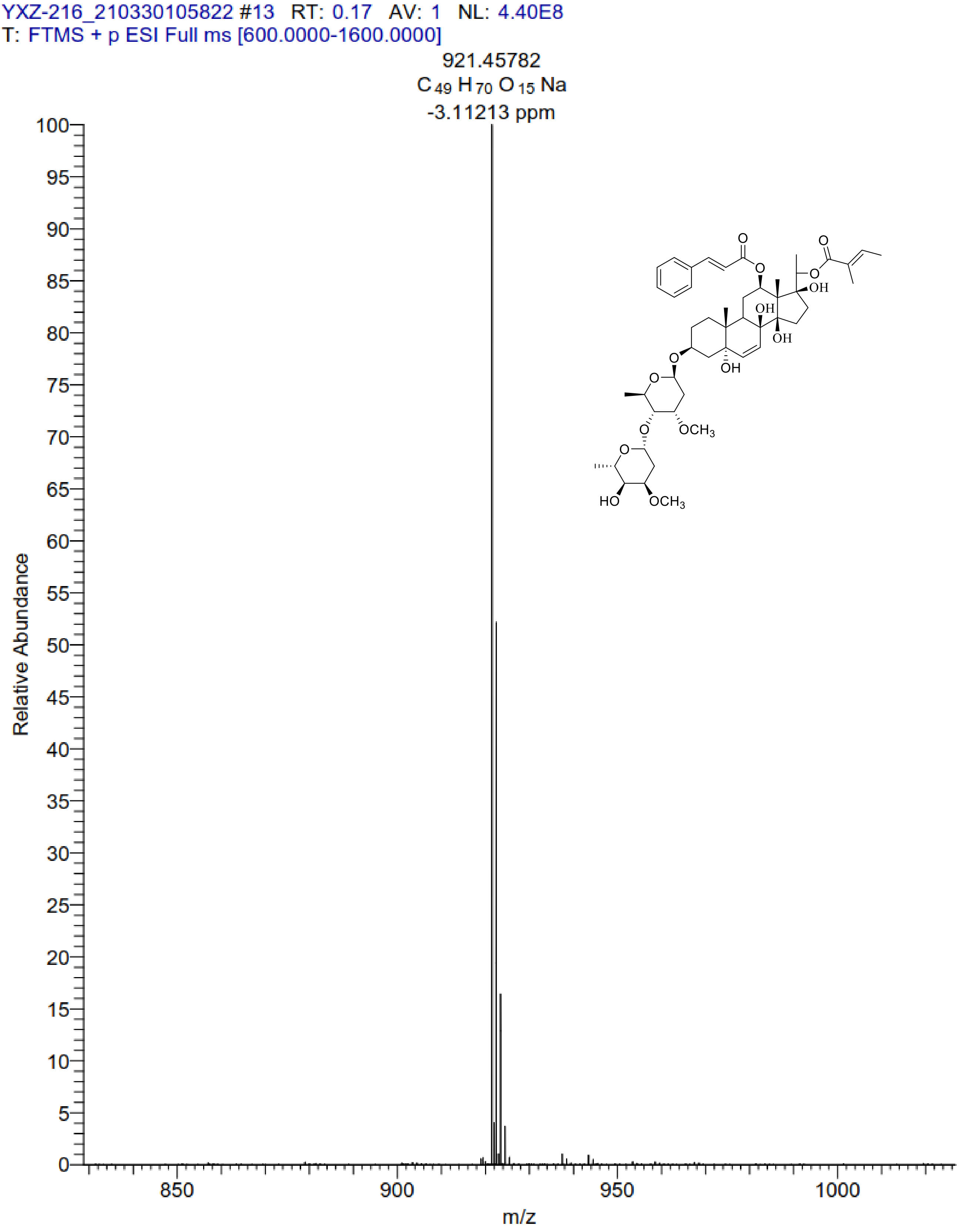

Figure S13 HRESIMS spectrum of **2**

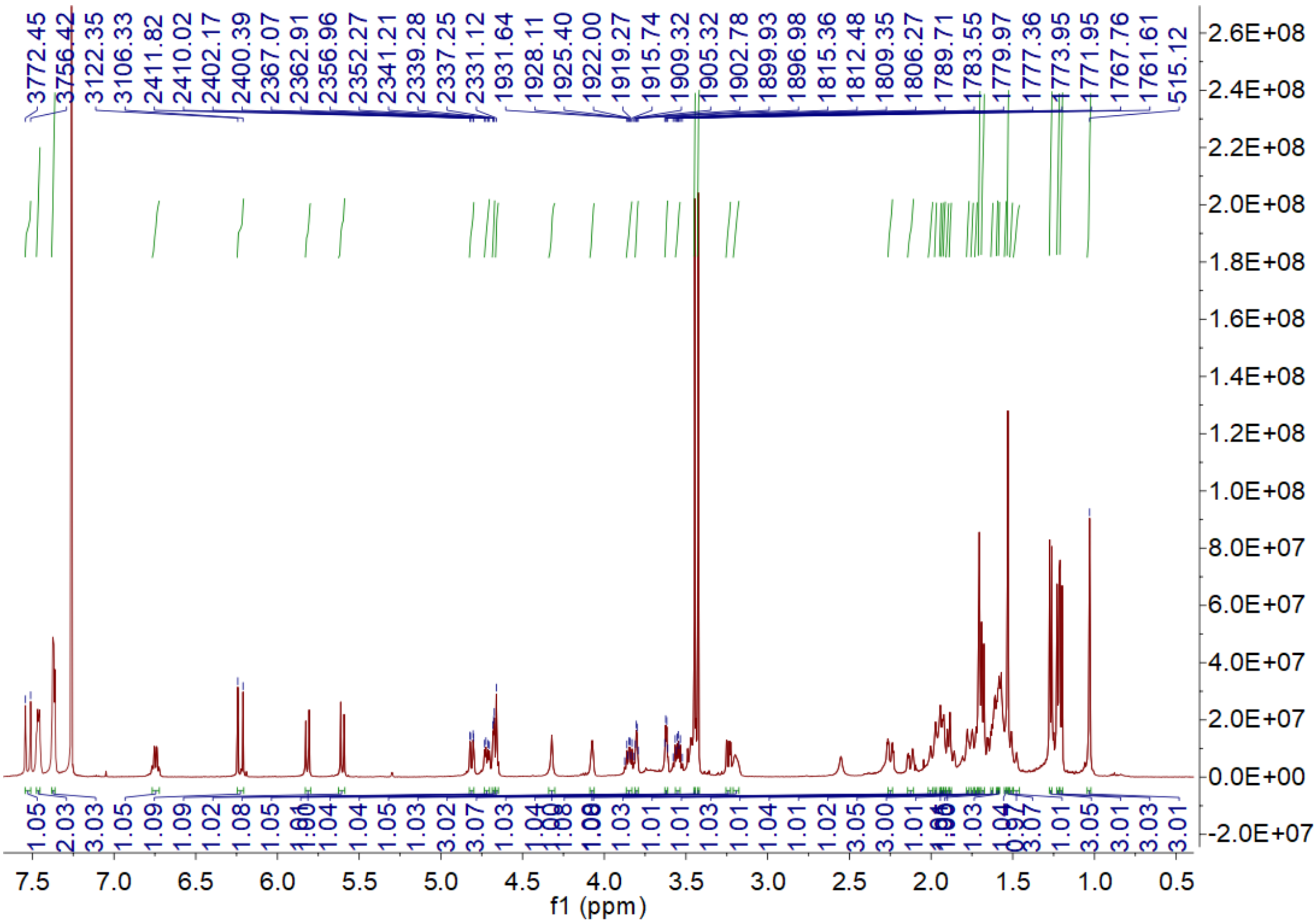

Figure S14 ^{1}H NMR spectrum (600 MHz, $CDCl_3$) of **2**

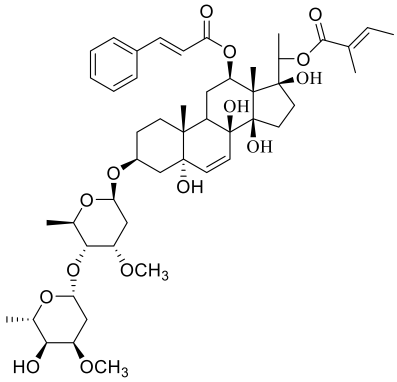

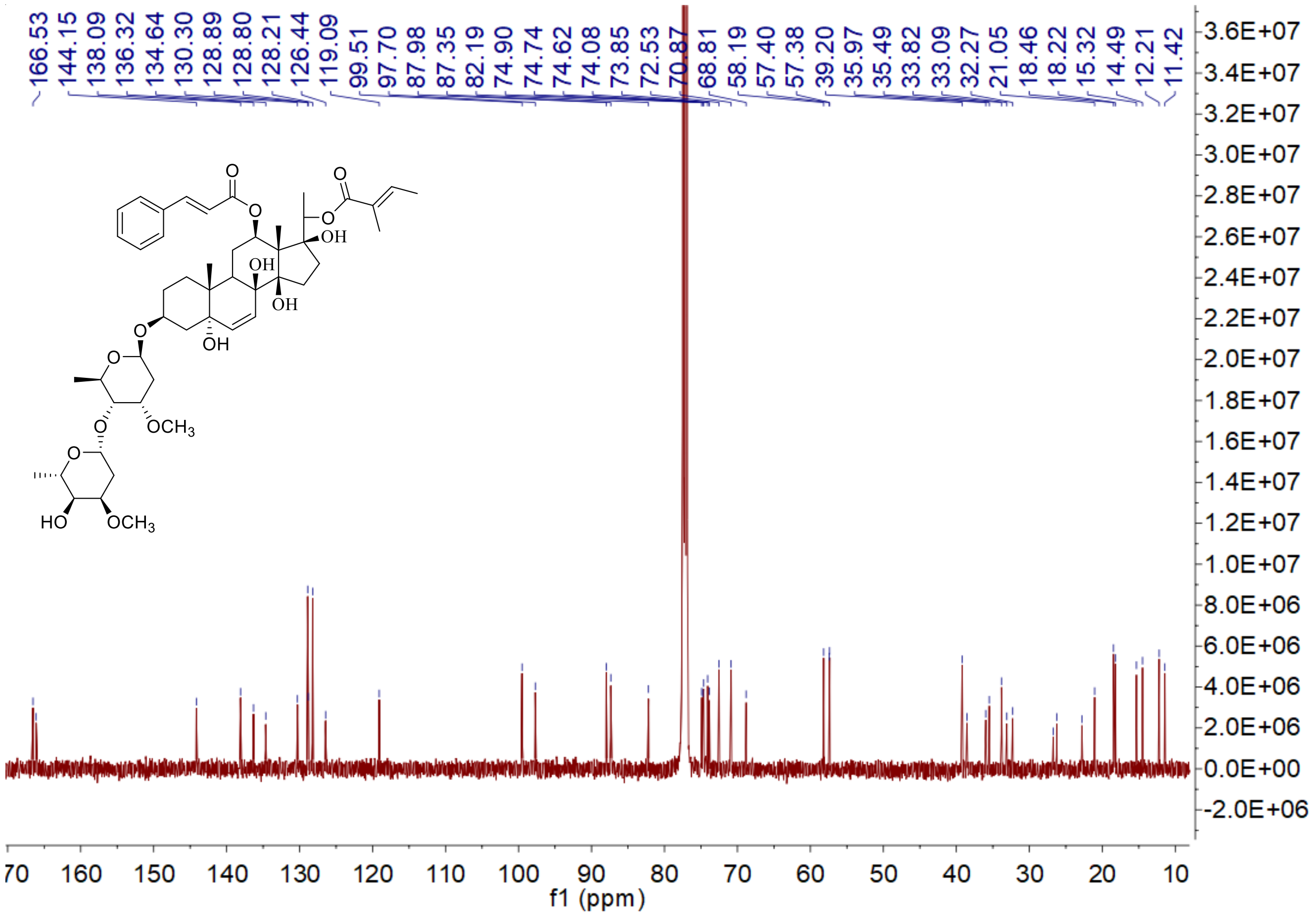

Fingue S15 ^{13}C NMR spectrum (150 MHz, $CDCl_3$) of **2**

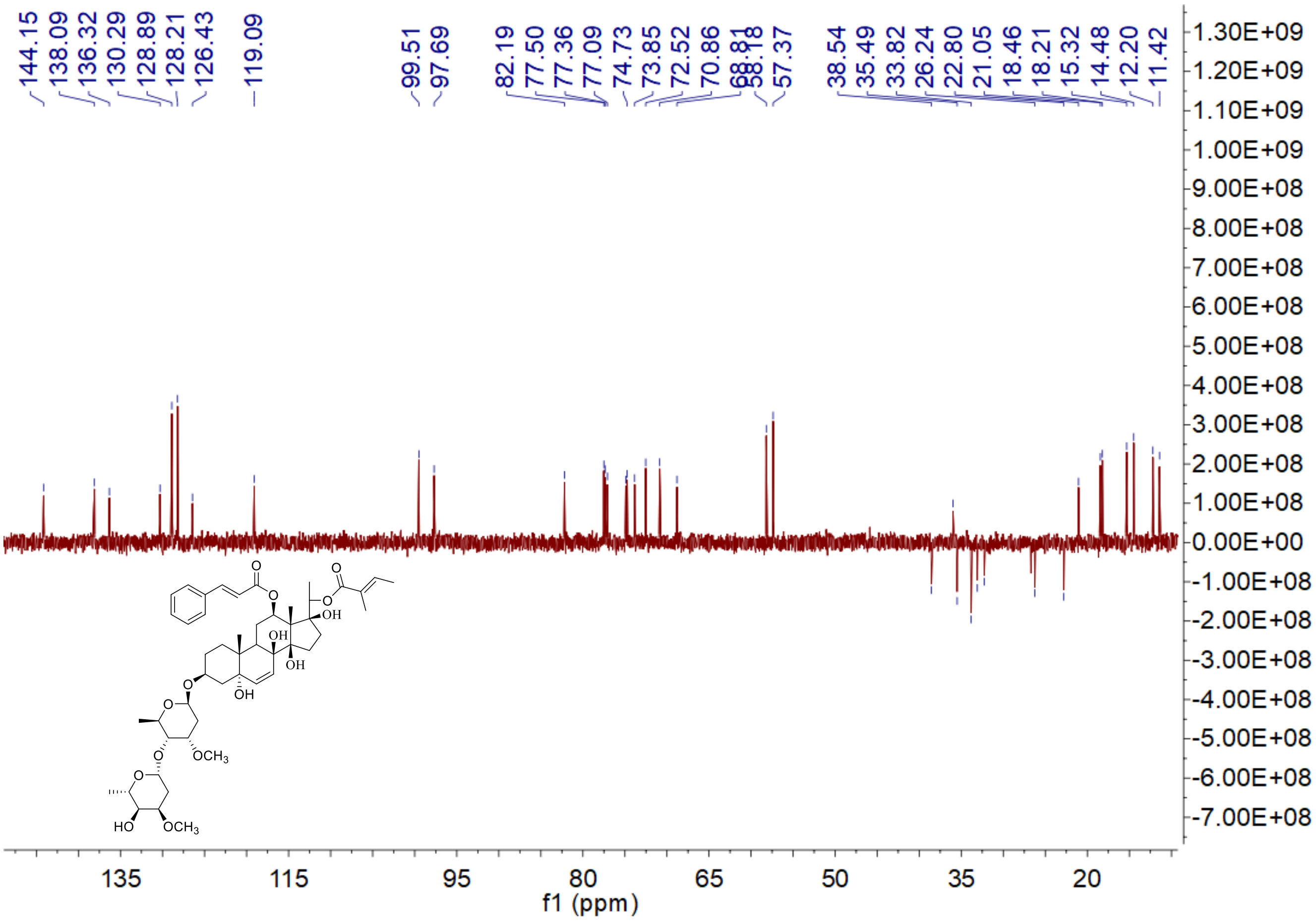

Figure S16 DEPT 135º spectrum (150 MHz, $CDCl_3$) of **2**

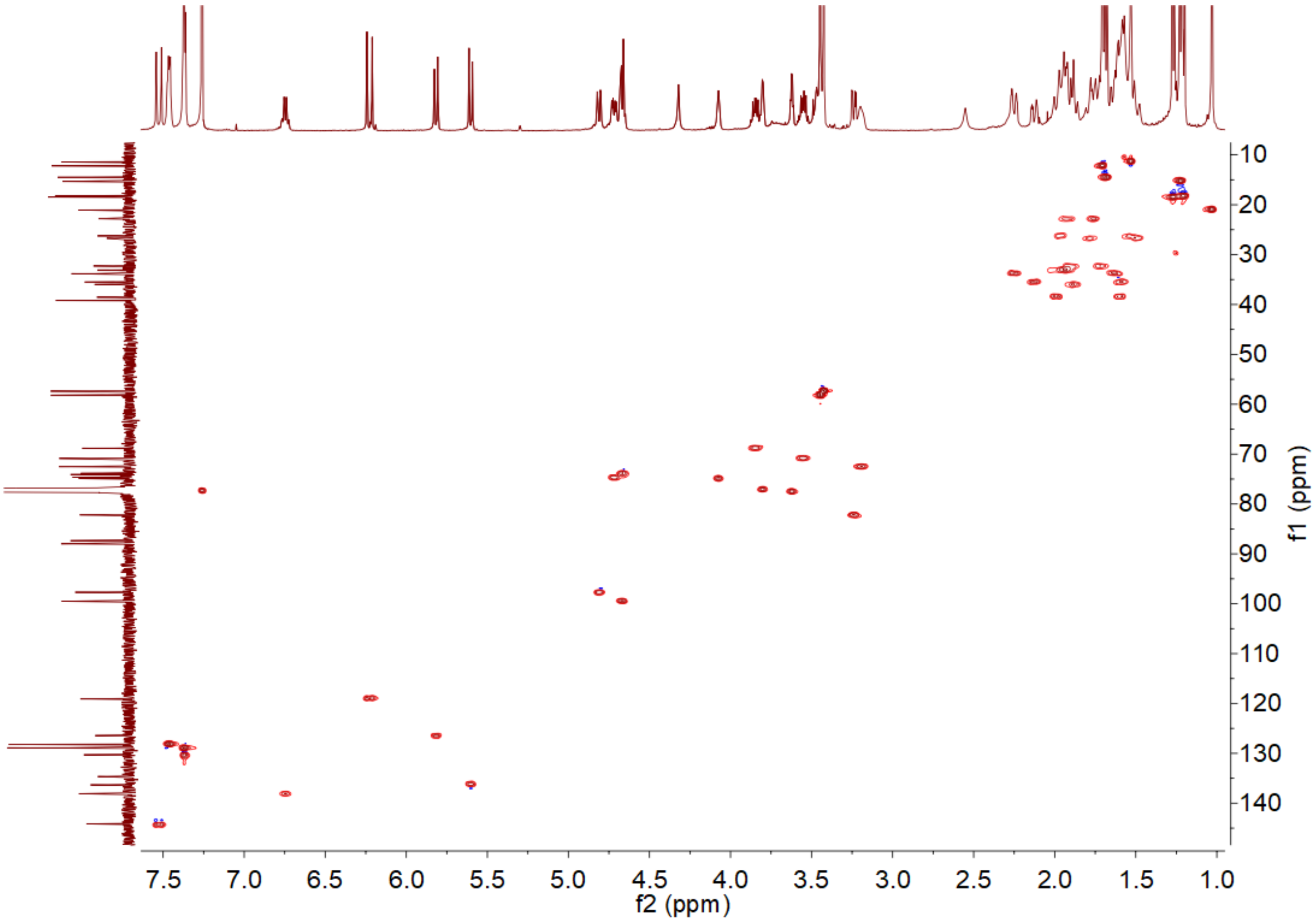

Figure S17 HSQC spectrum of **2**

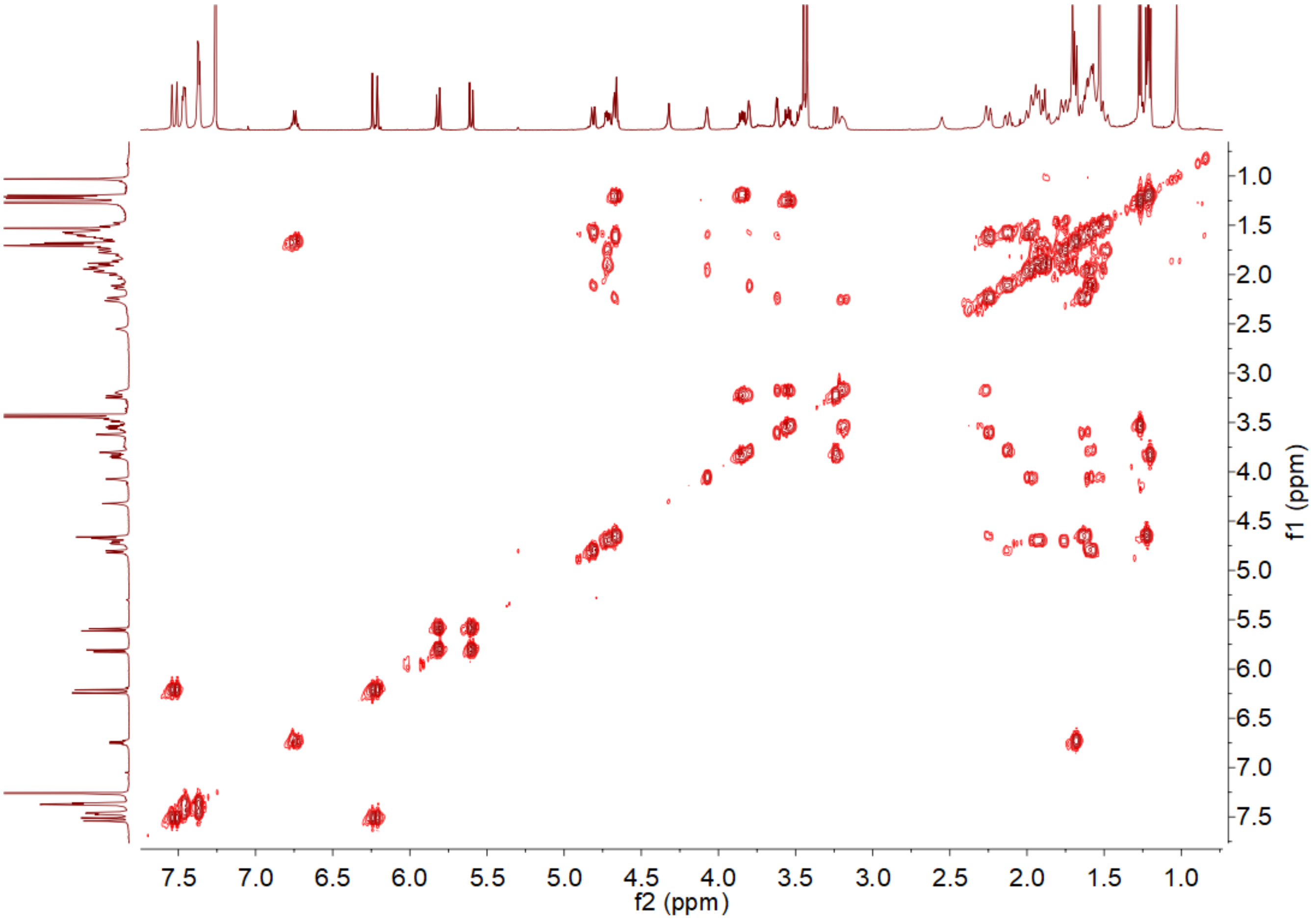

Figure S18 COSY spectrum of **2**

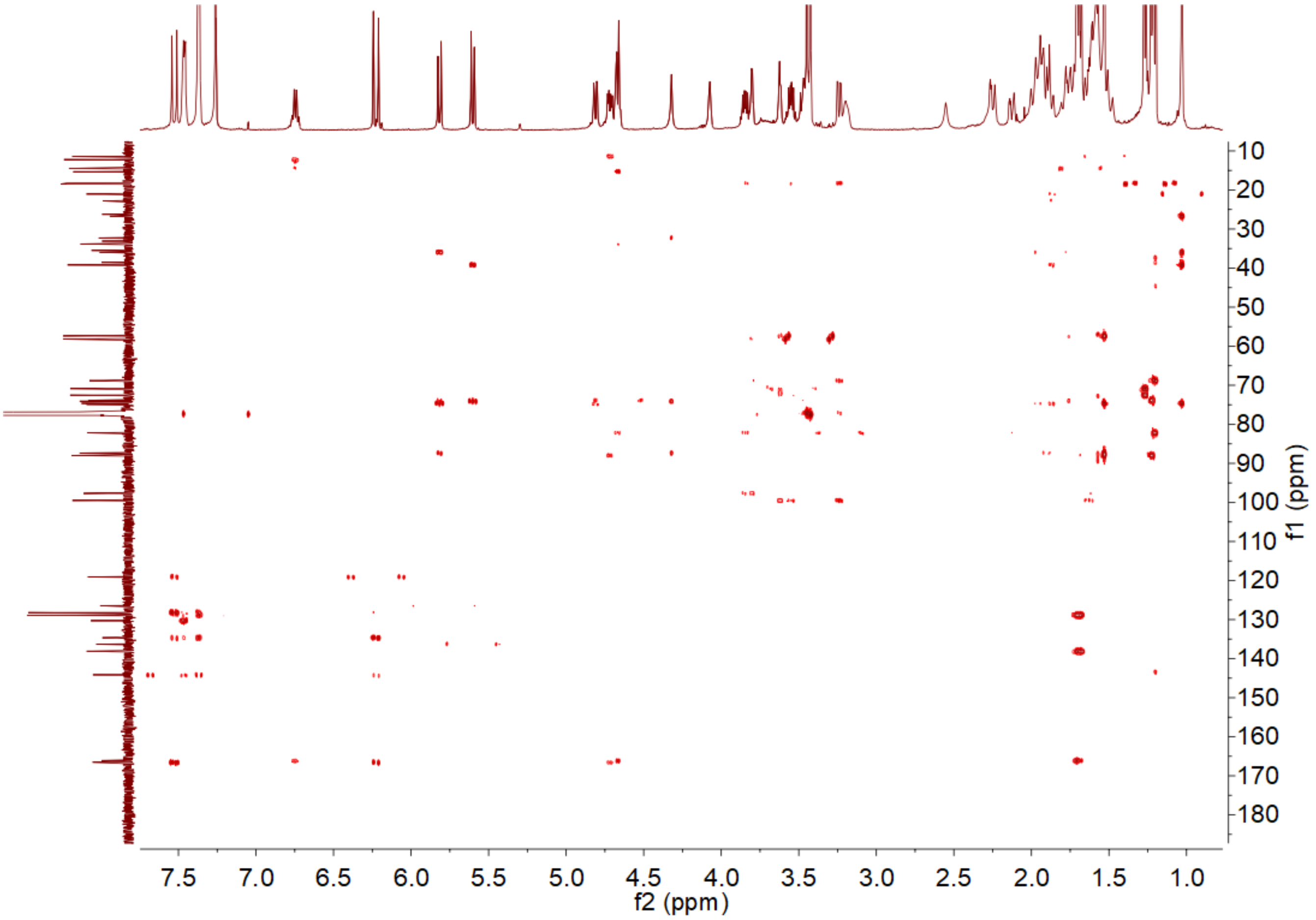

Figure S19 HMBC spectrum of **2**

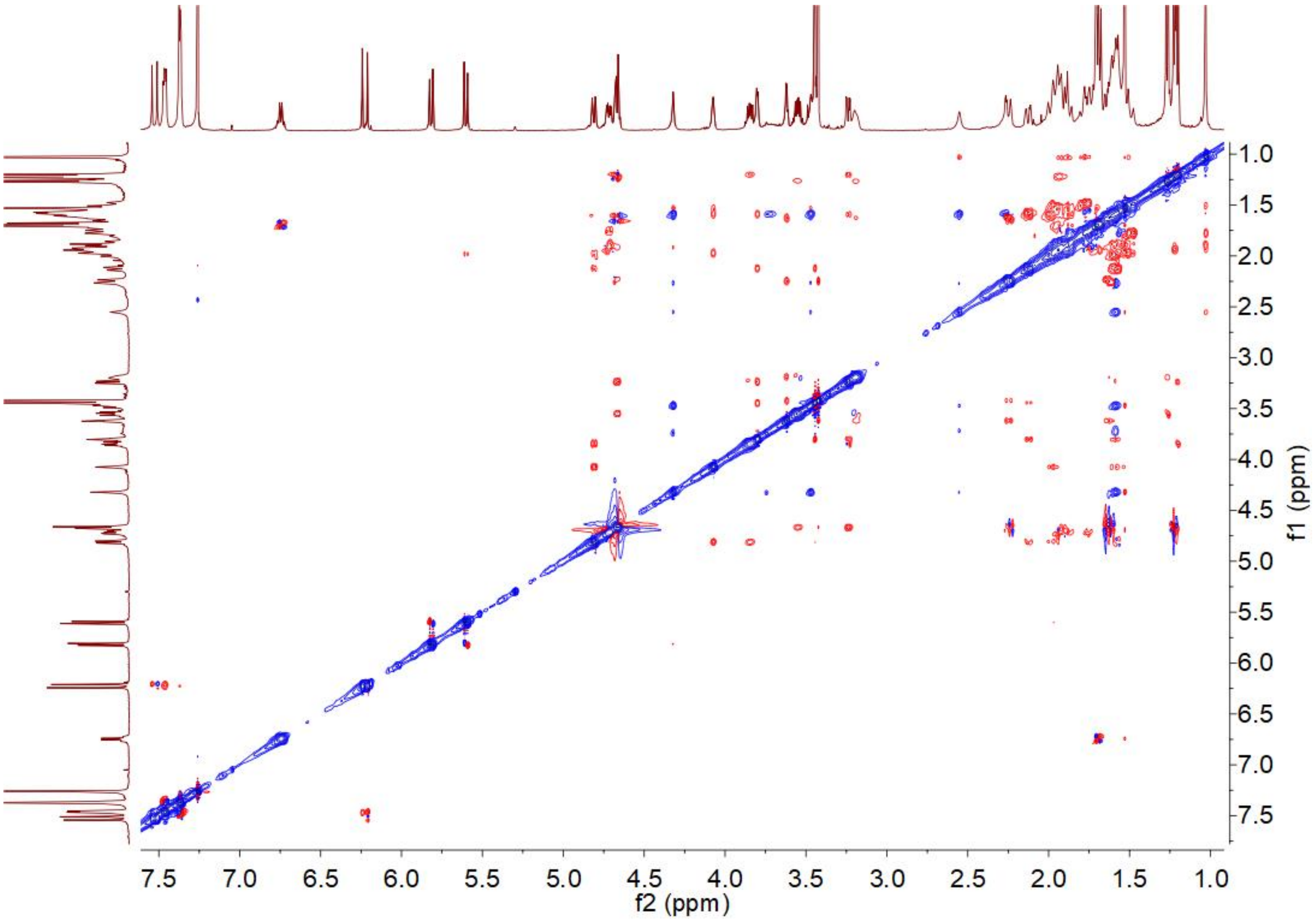

Figure S20 NOESY spectrum of **2**

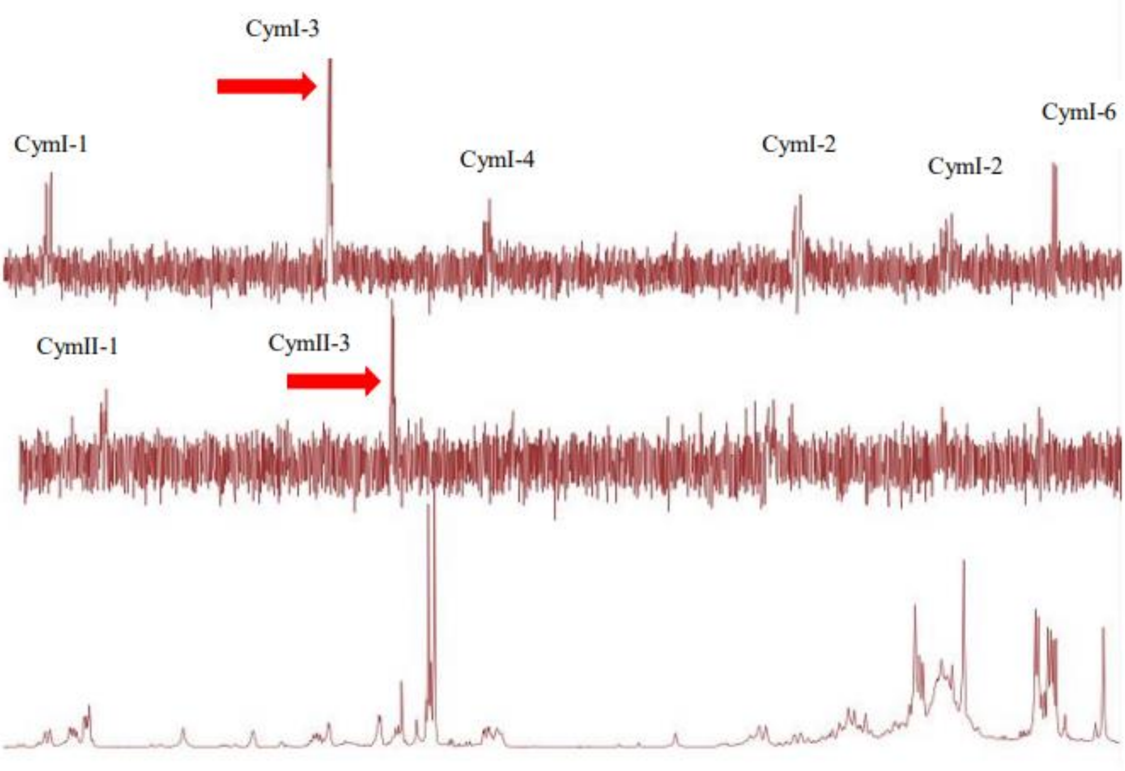

Figure S21 1D TOCSY spectrum of **2**

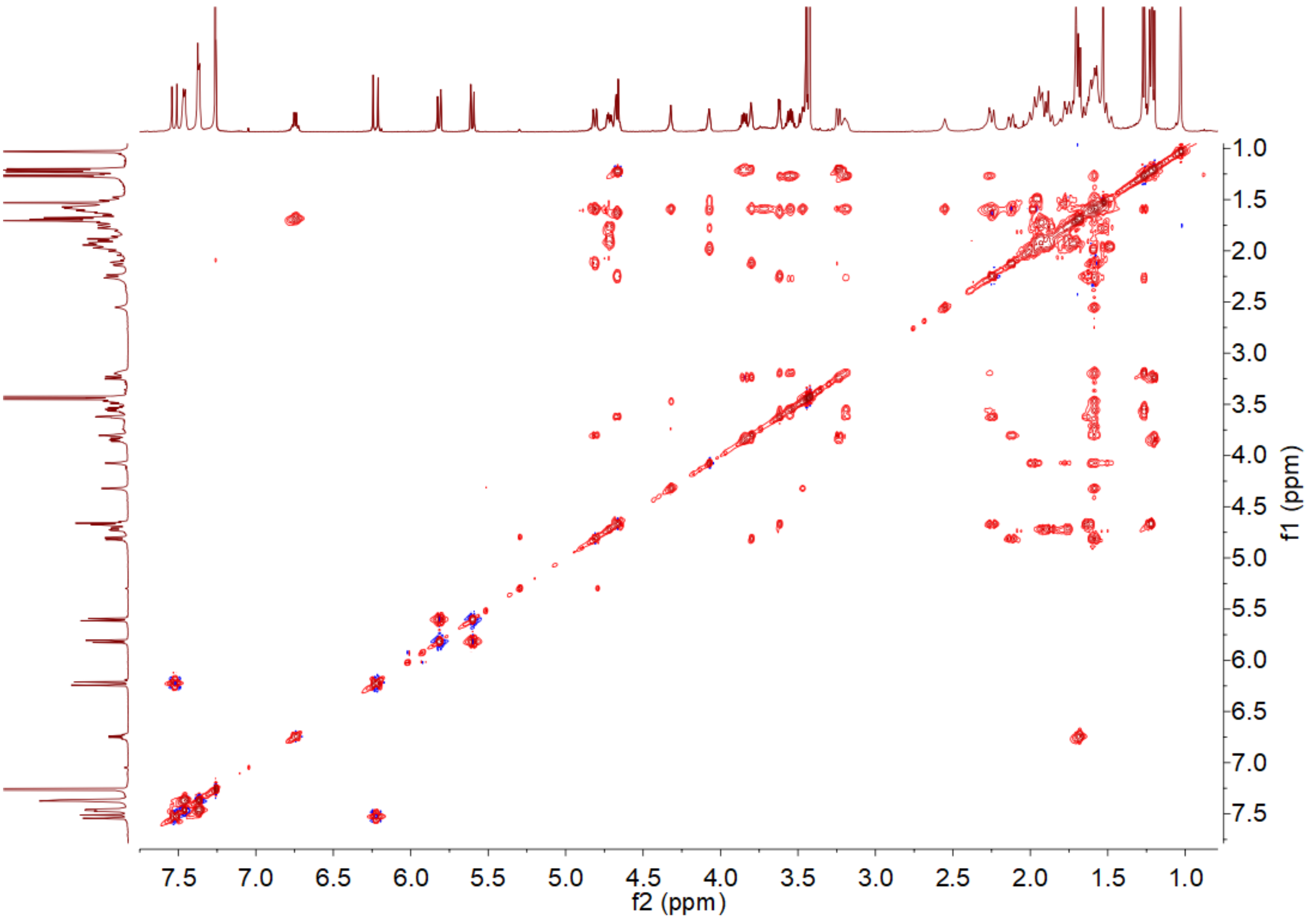

Figure S22 2D TOCSY spectrum of **2**

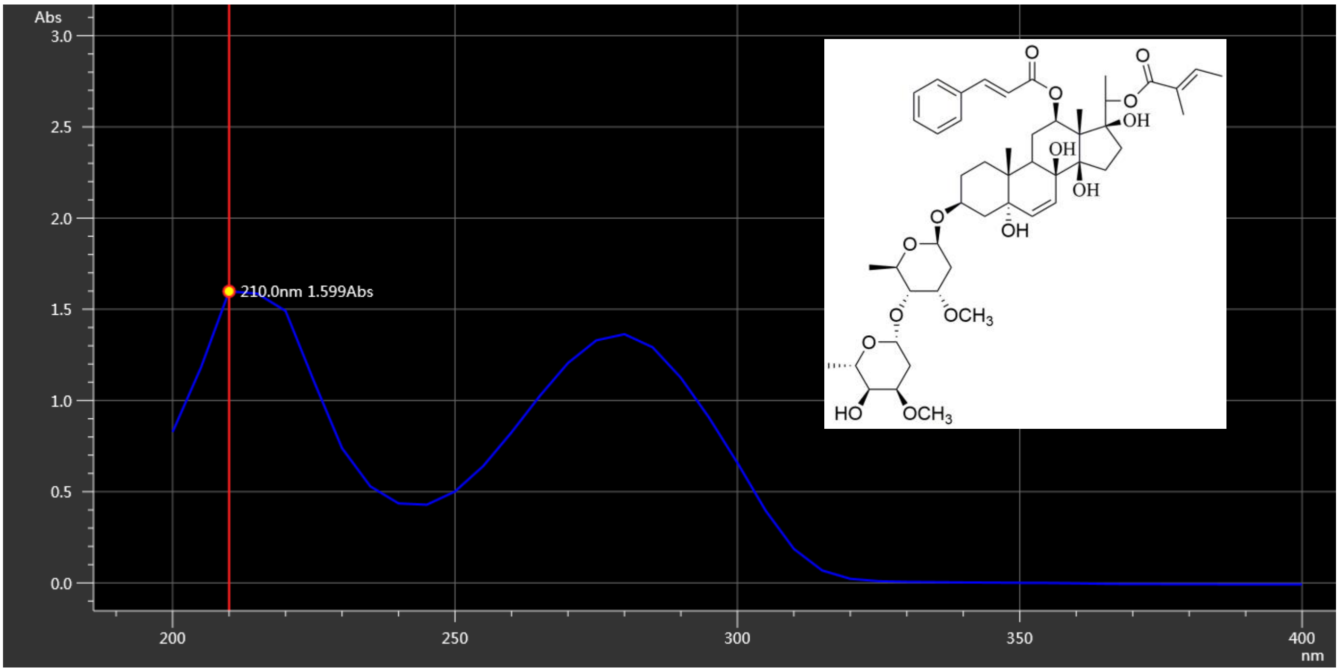

Figure S23 UV spectrum of **2**

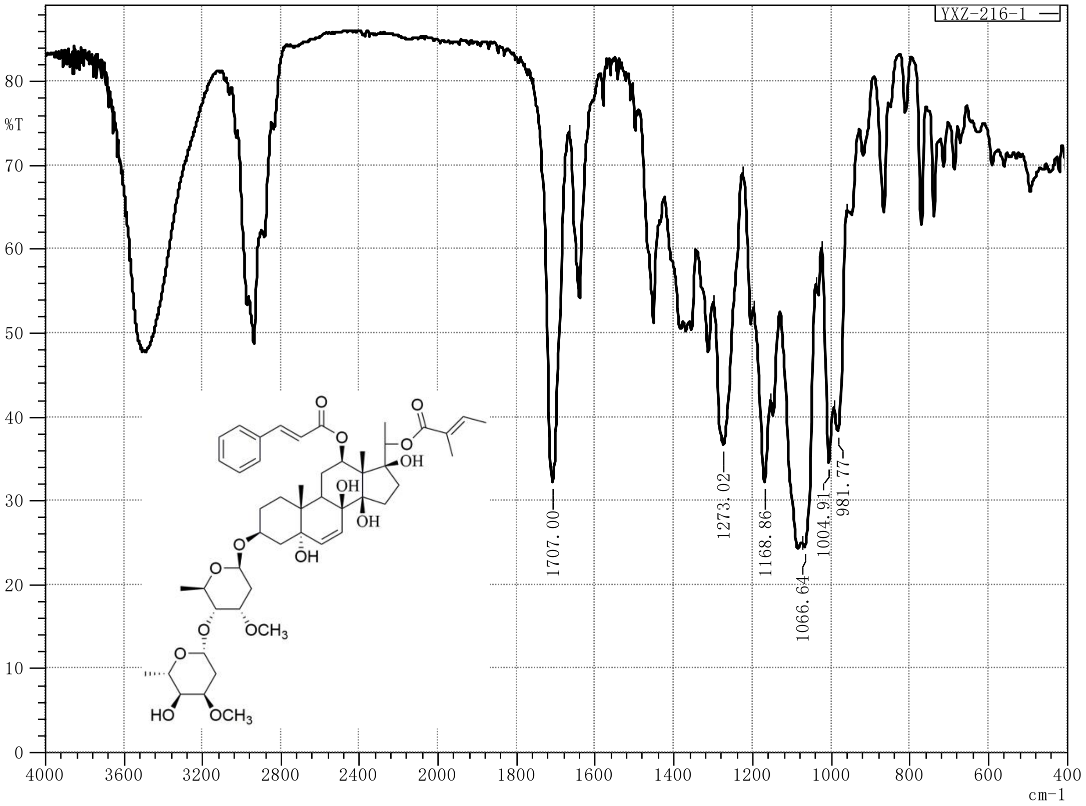

Figure S24 IR spectrum of **2**

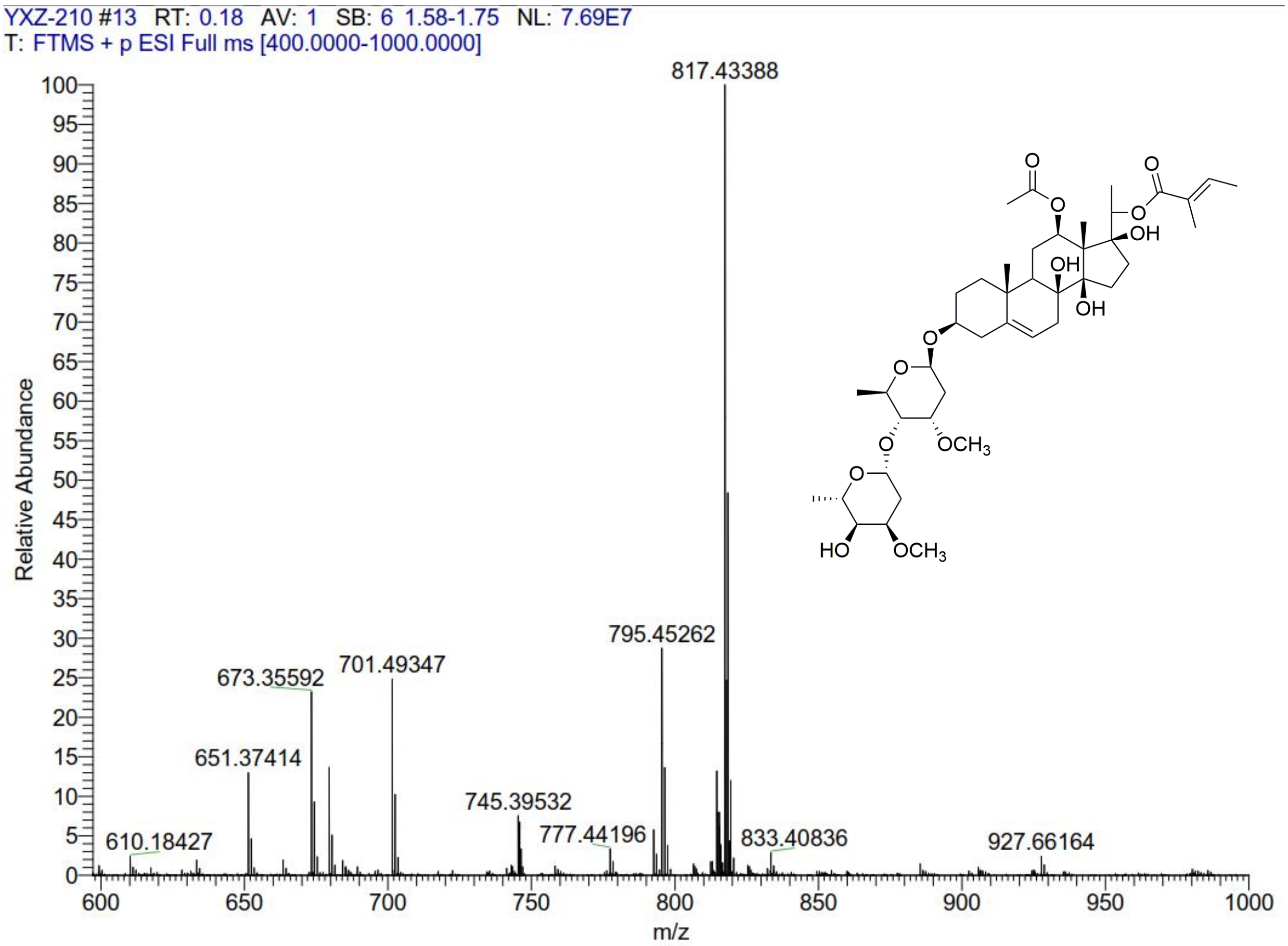

Figure S25 HRESIMS spectrum of **3**

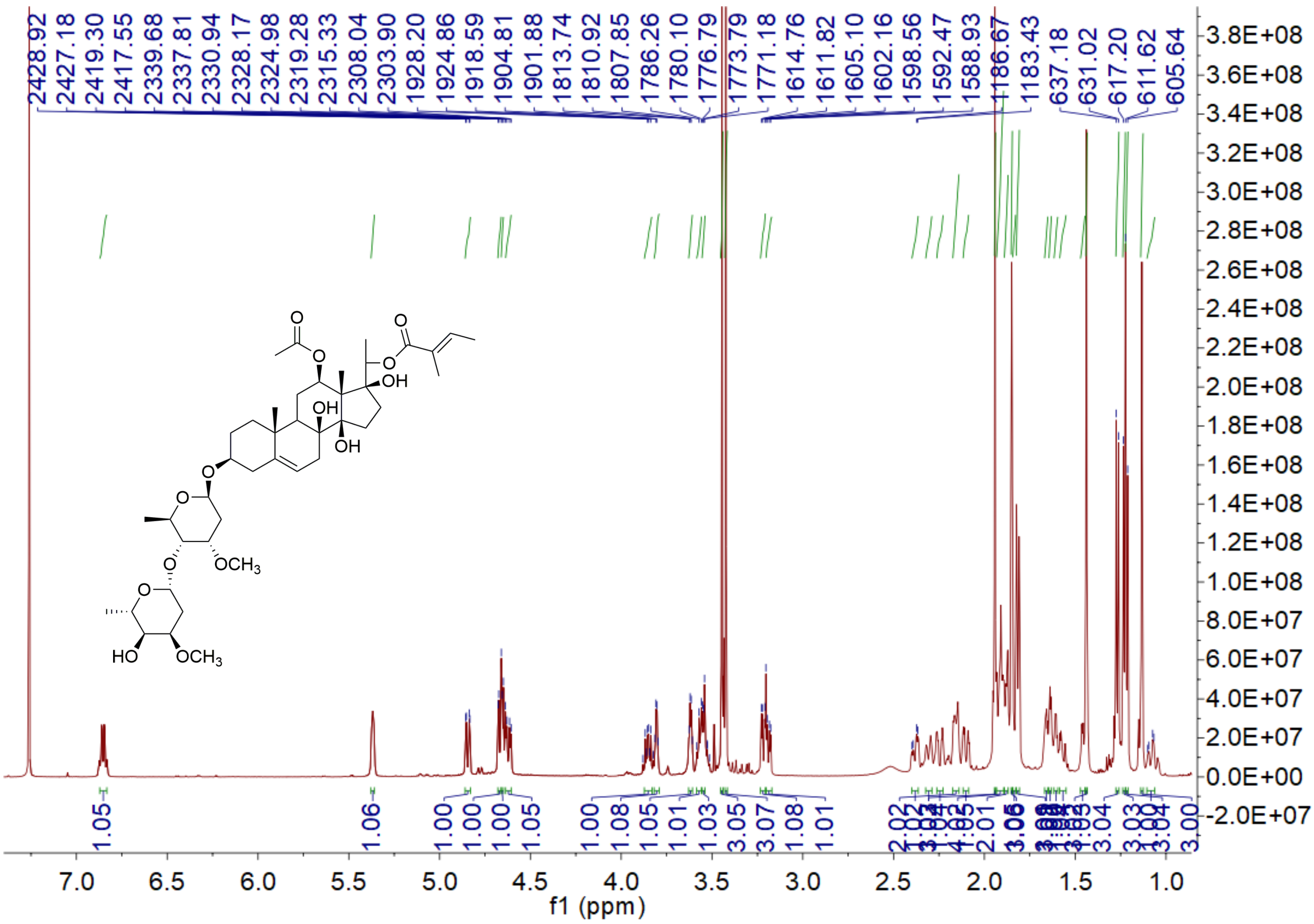

Figure S26 ^{1}H NMR spectrum (600 MHz, $CDCl_3$) of **3**

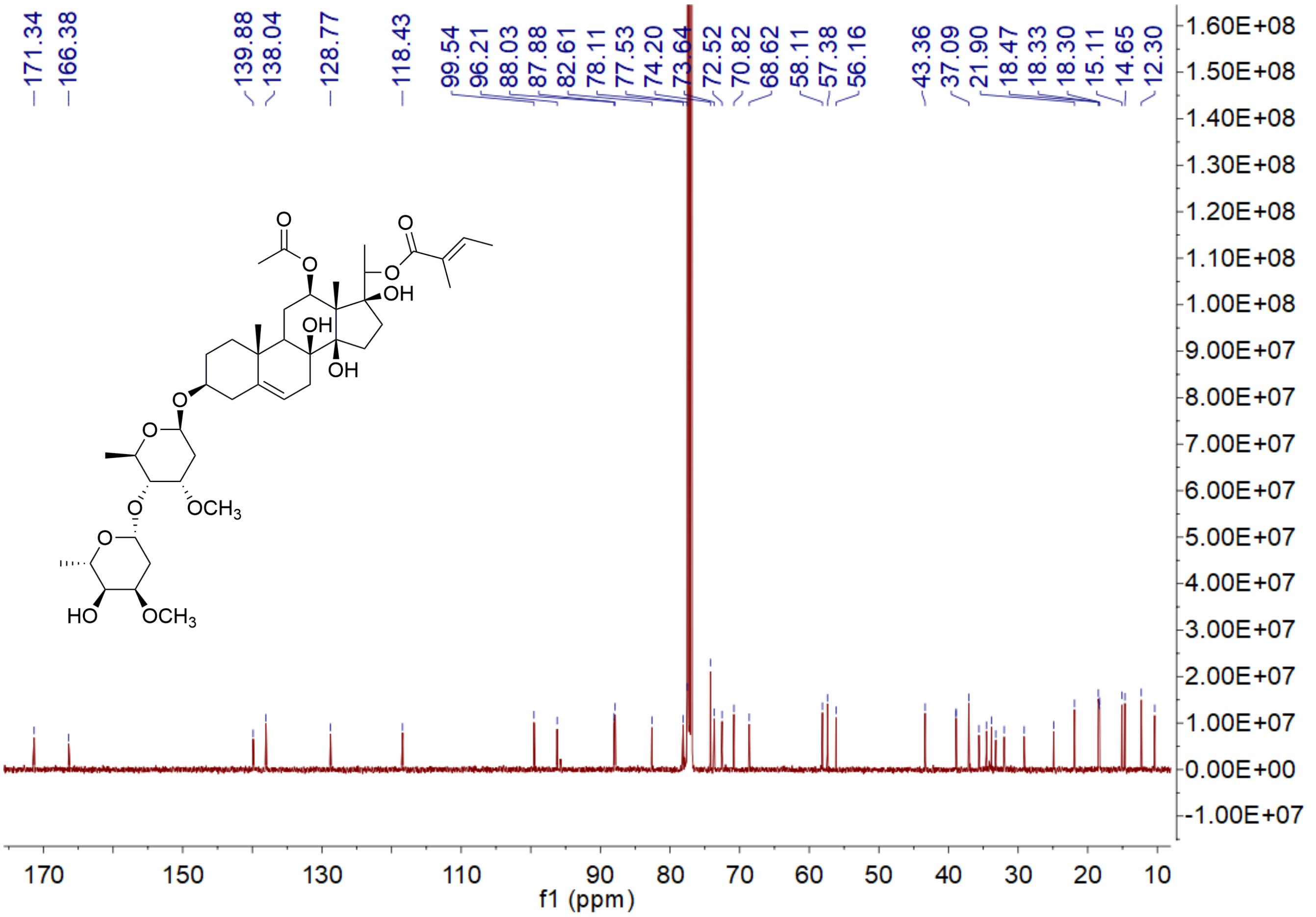

Figure S27 ^{13}C NMR spectrum (150 MHz, $CDCl_3$) of **3**

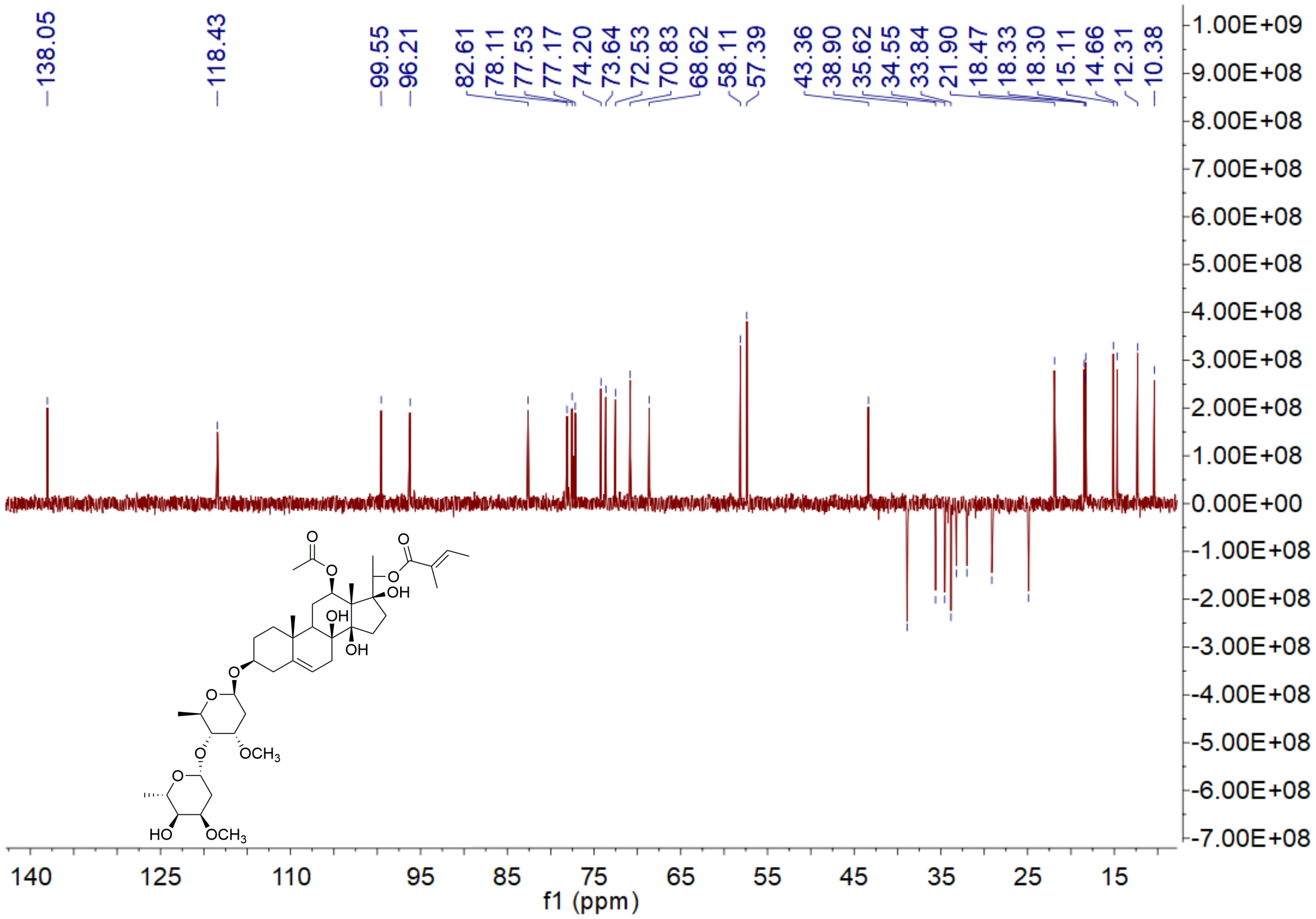

Figure S28 DEPT 135º spectrum (150 MHz, $CDCl_3$) of **3**

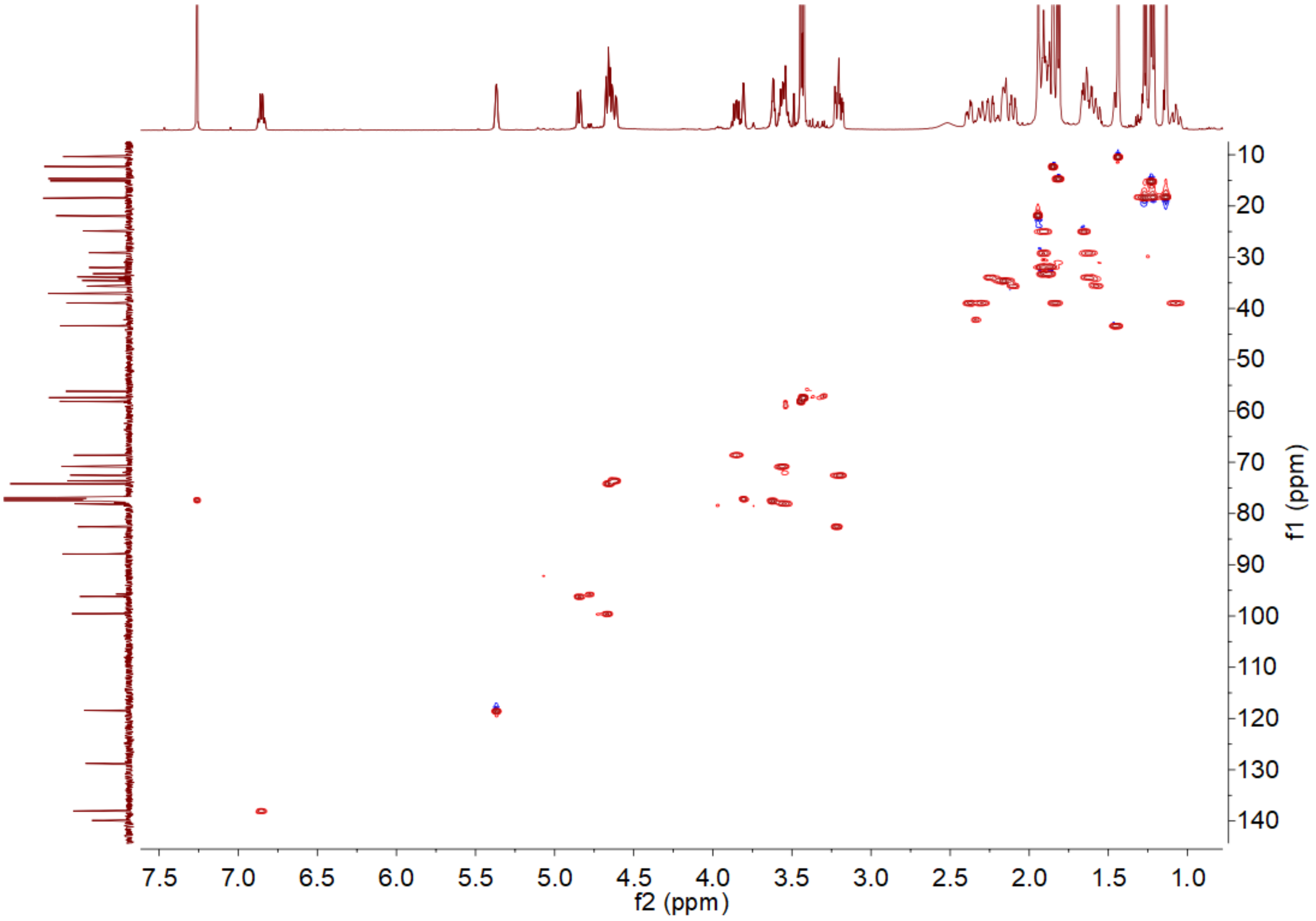

Figure S29 HSQC spectrum of **3**

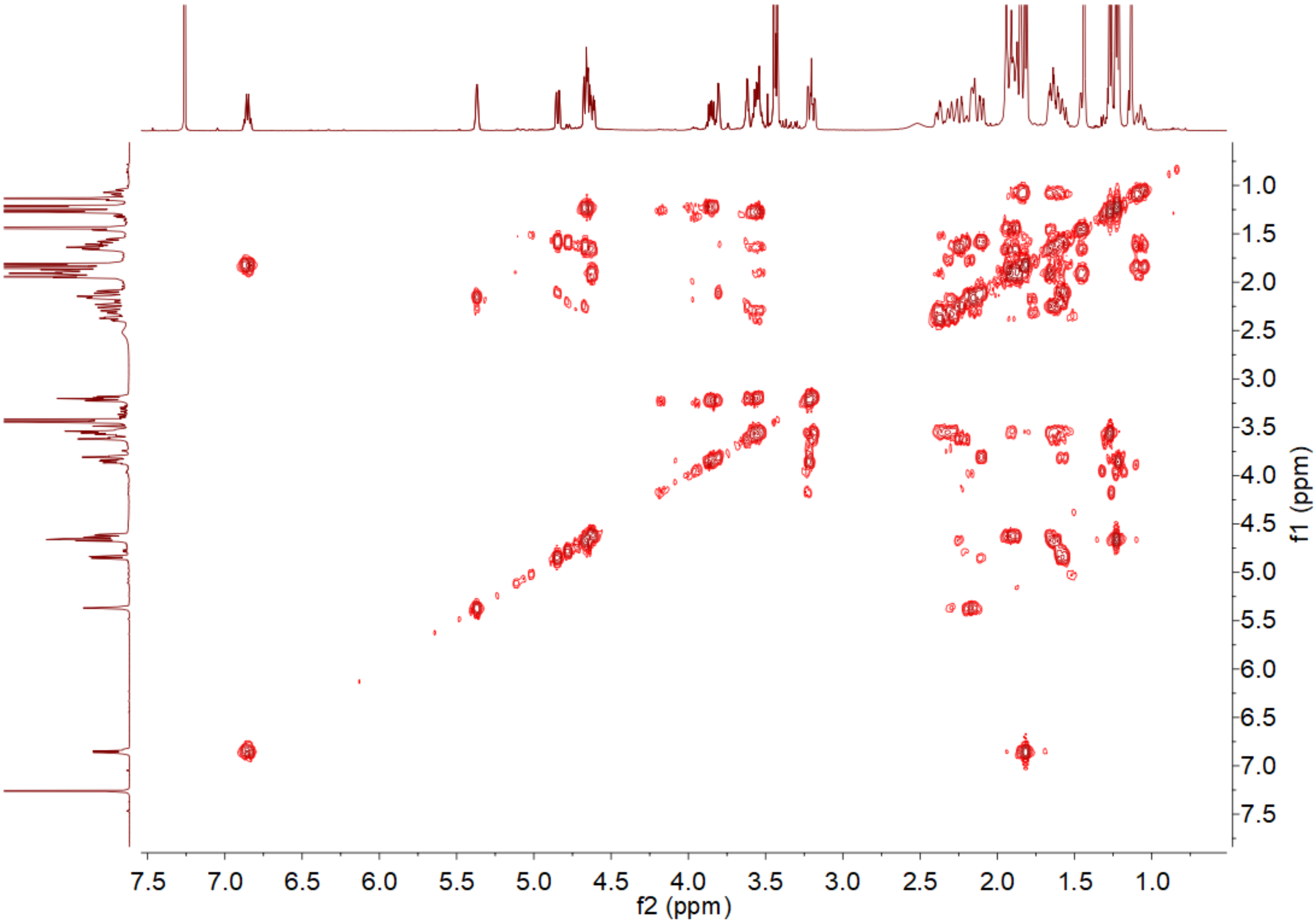

Figure S30 COSY spectrum of **3**

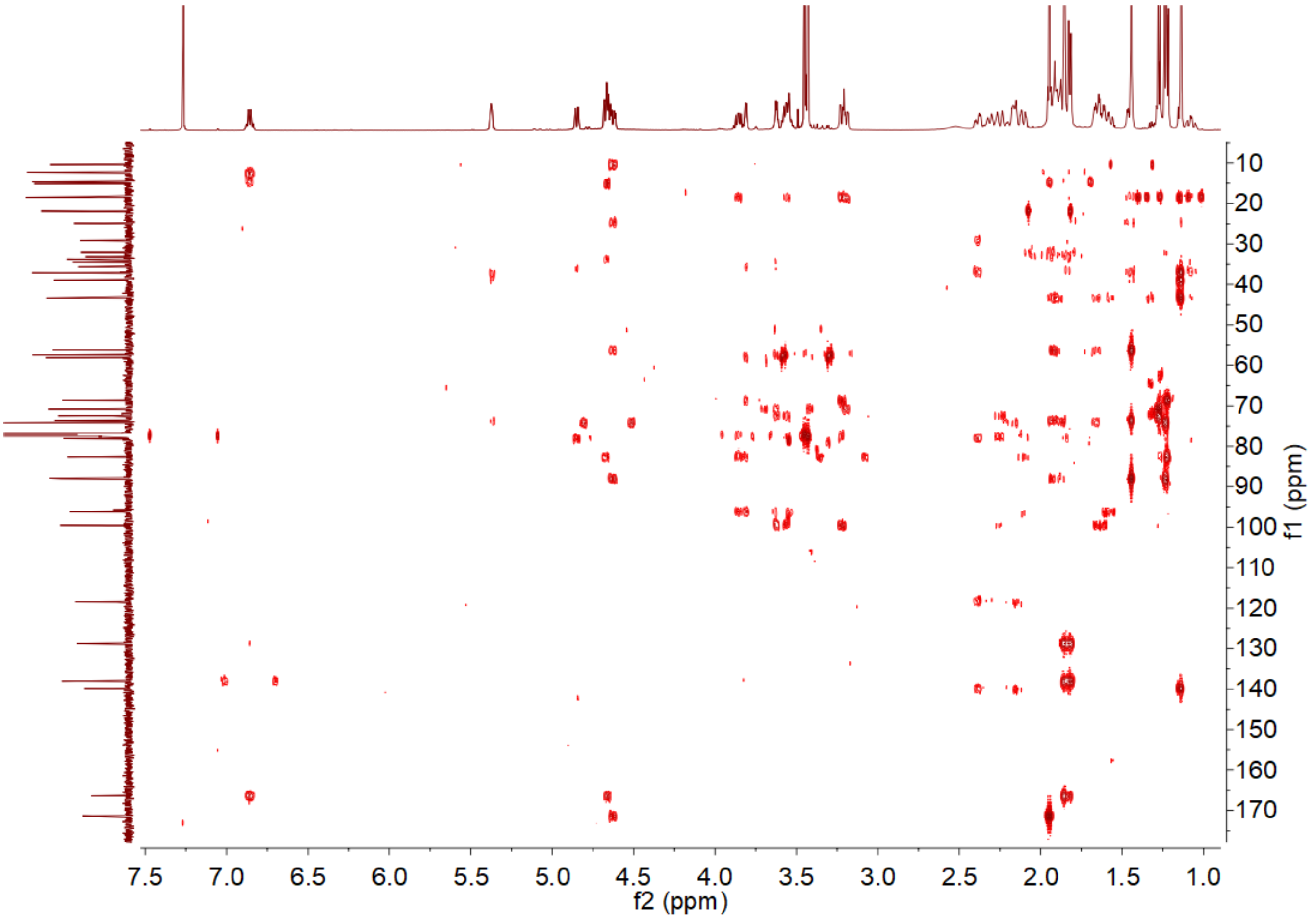

Figure S31 HMBC spectrum of **3**

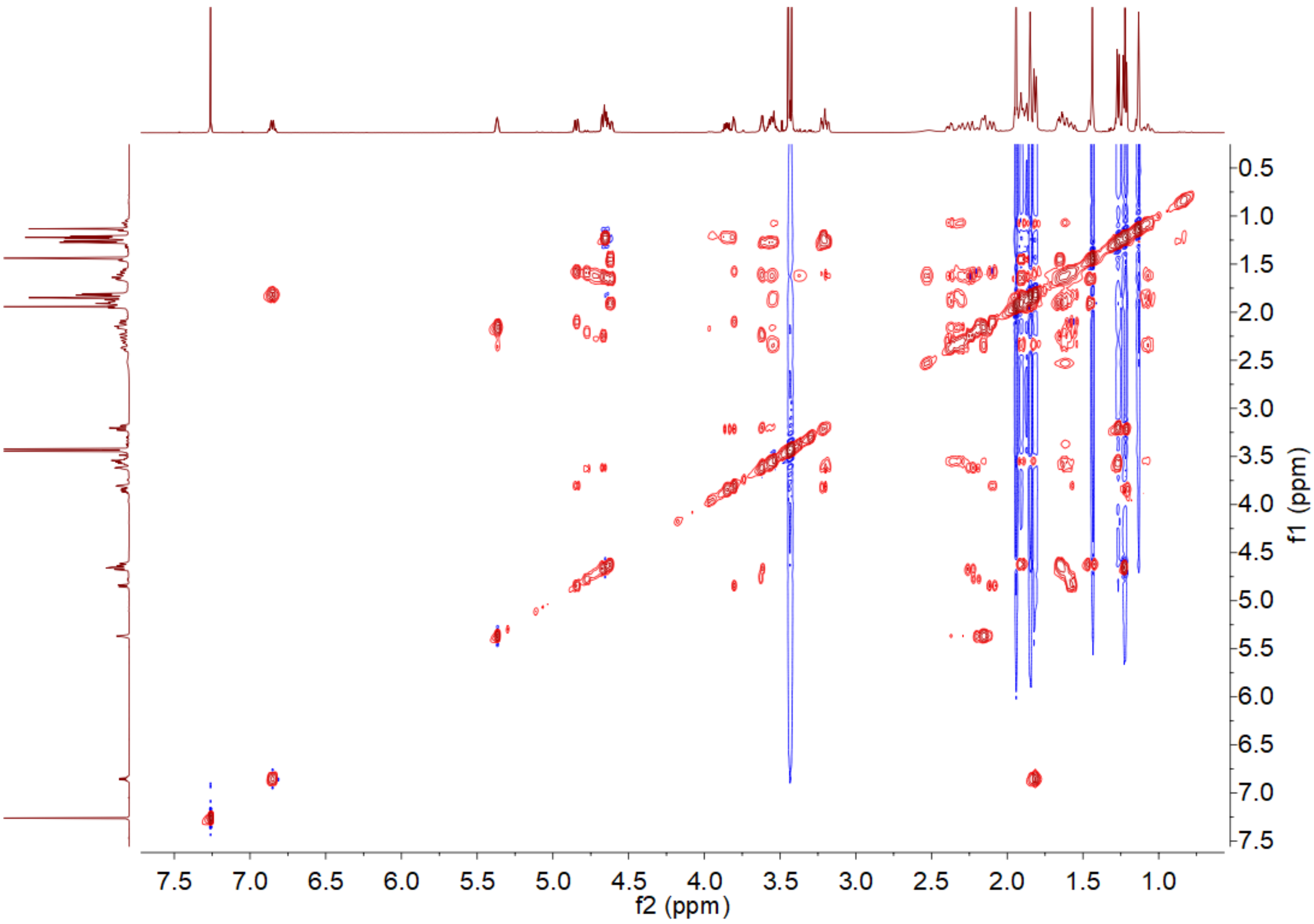

Figure S32 NOESY spectrum of **3**

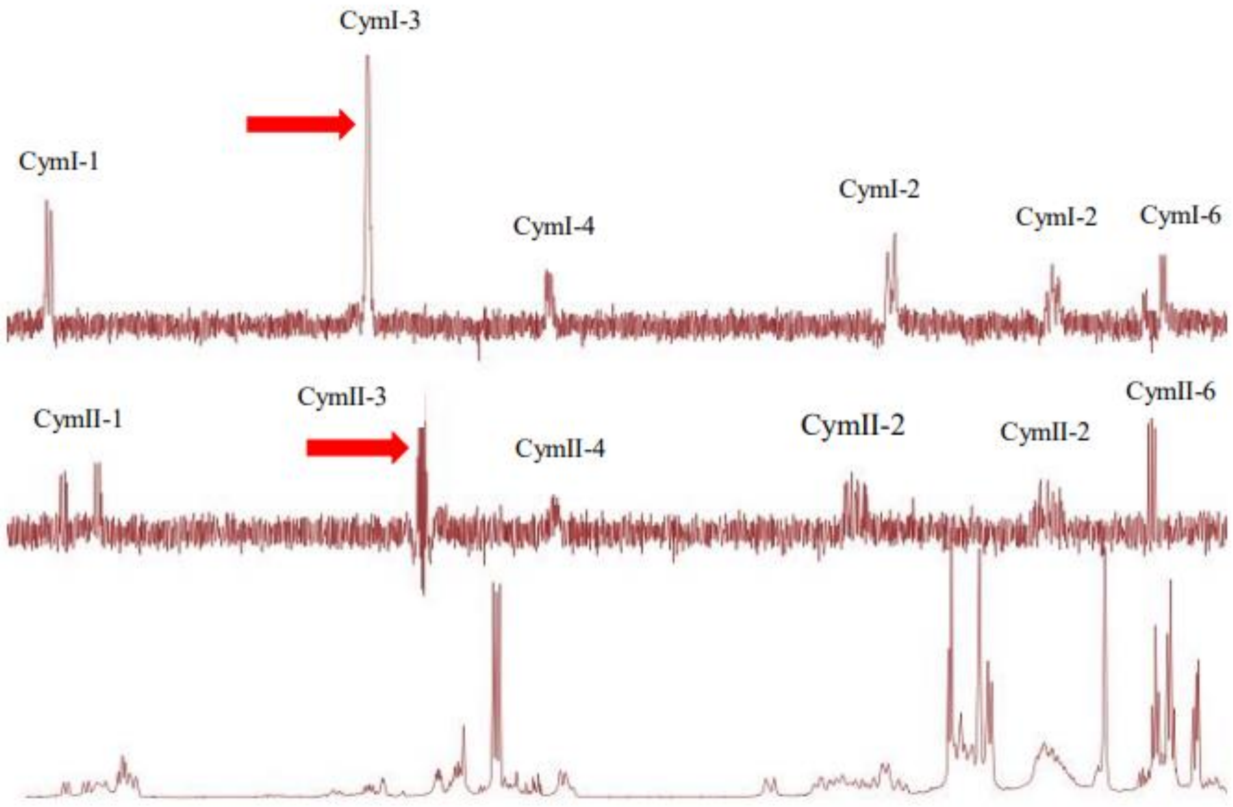

Figure S33 1D TOCSY spectrum of **3**

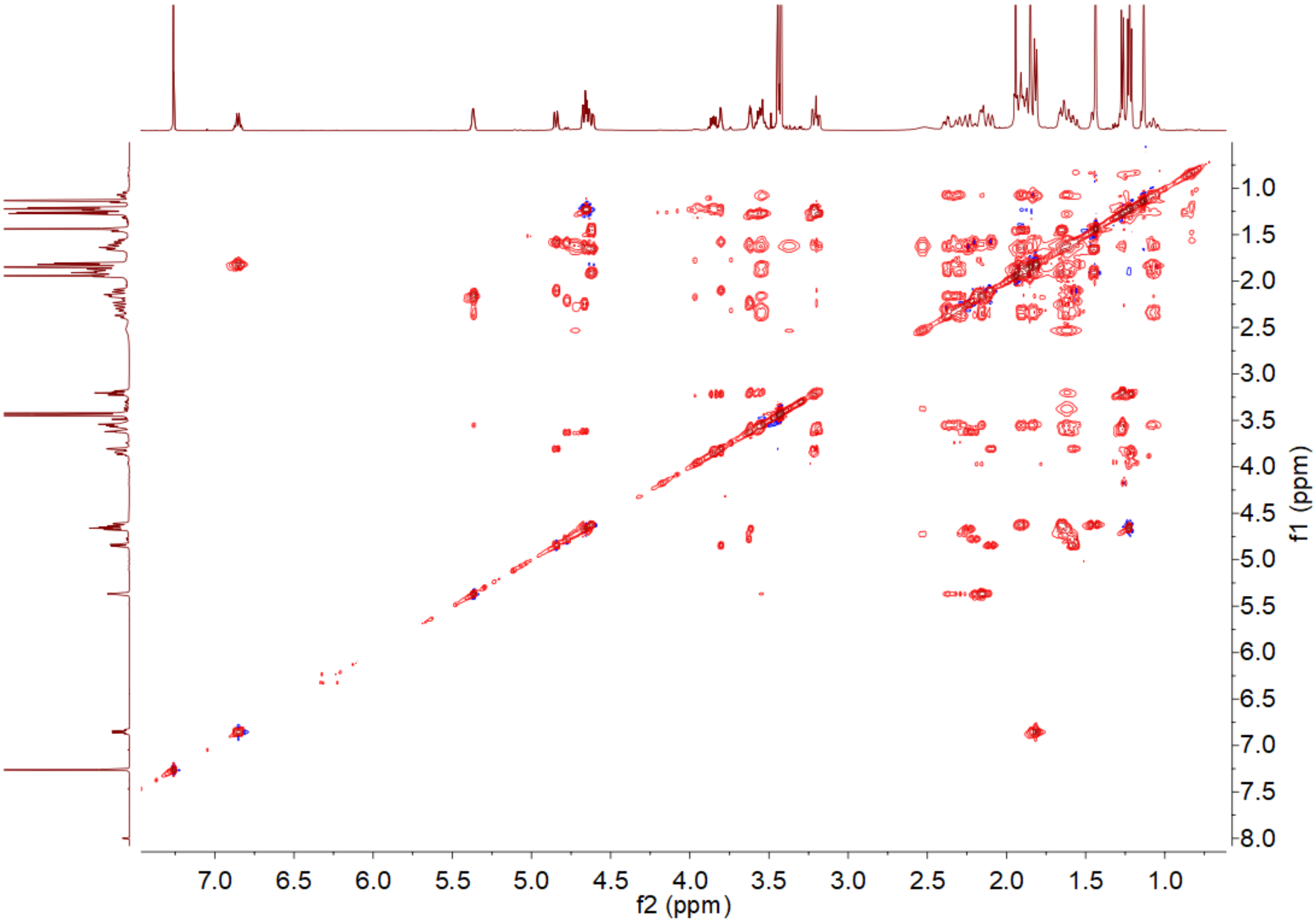

Figure S34 2D TOCSY spectrum of **3**

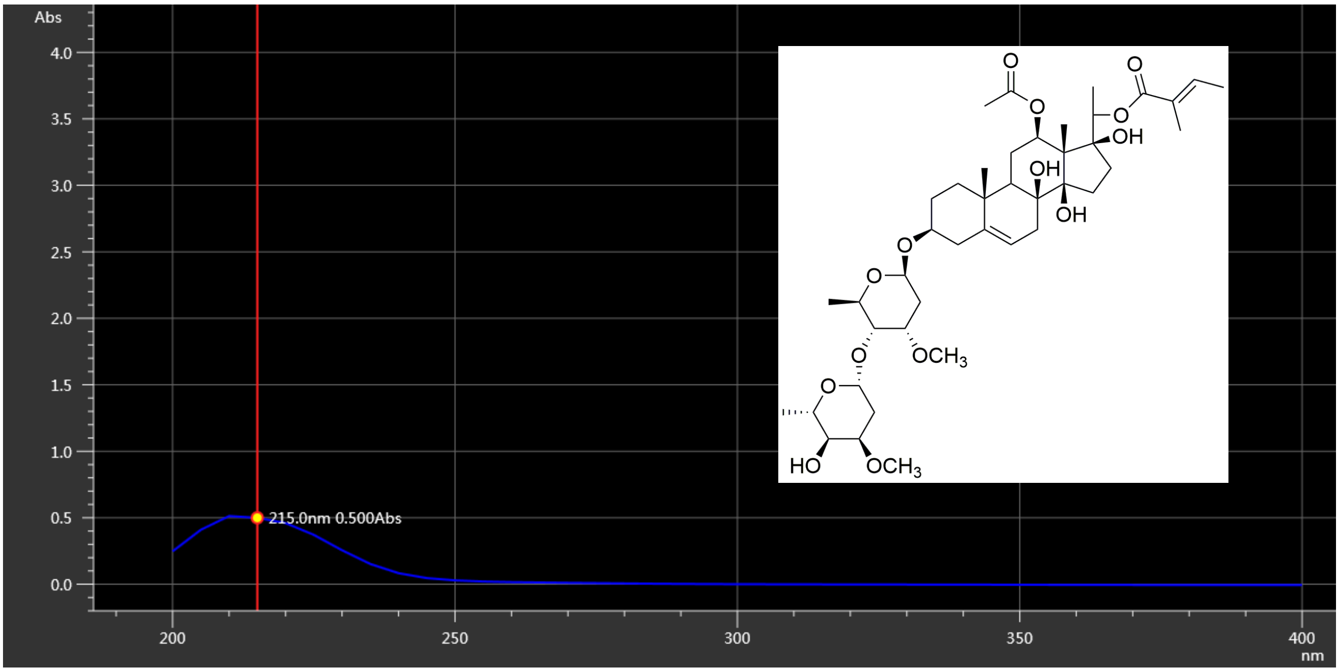

Figure S35 UV spectrum of **3**

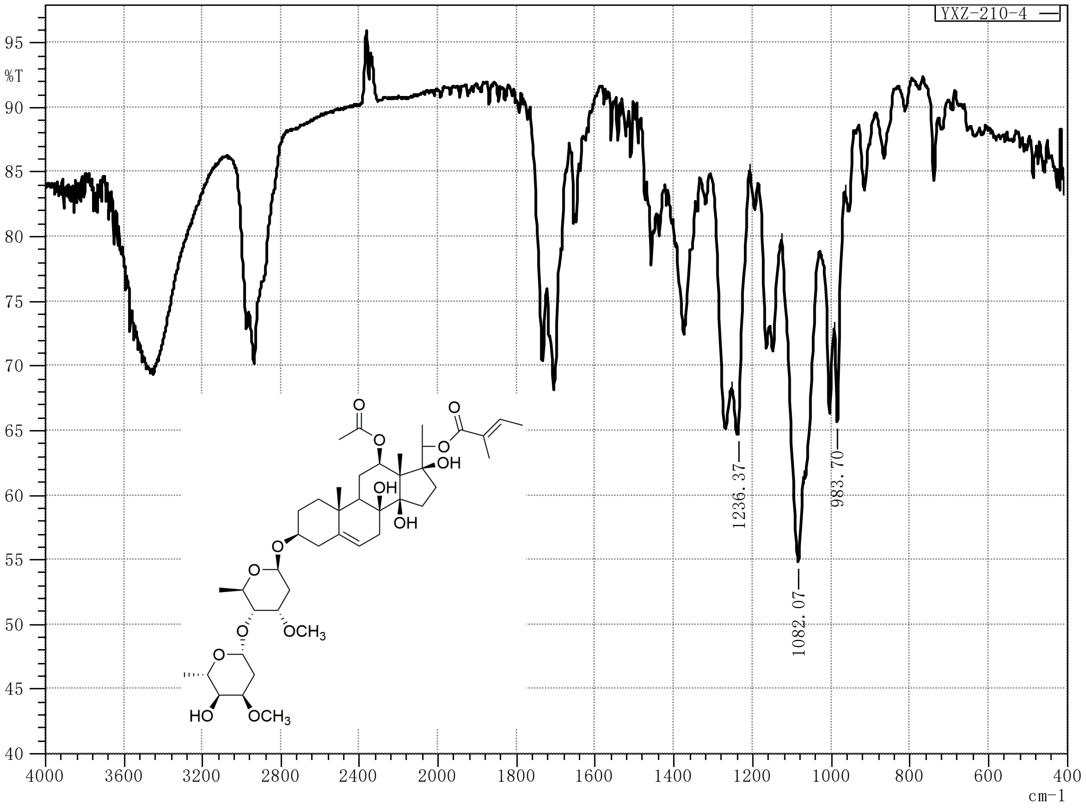

Figure S36 IR spectrum of **3**

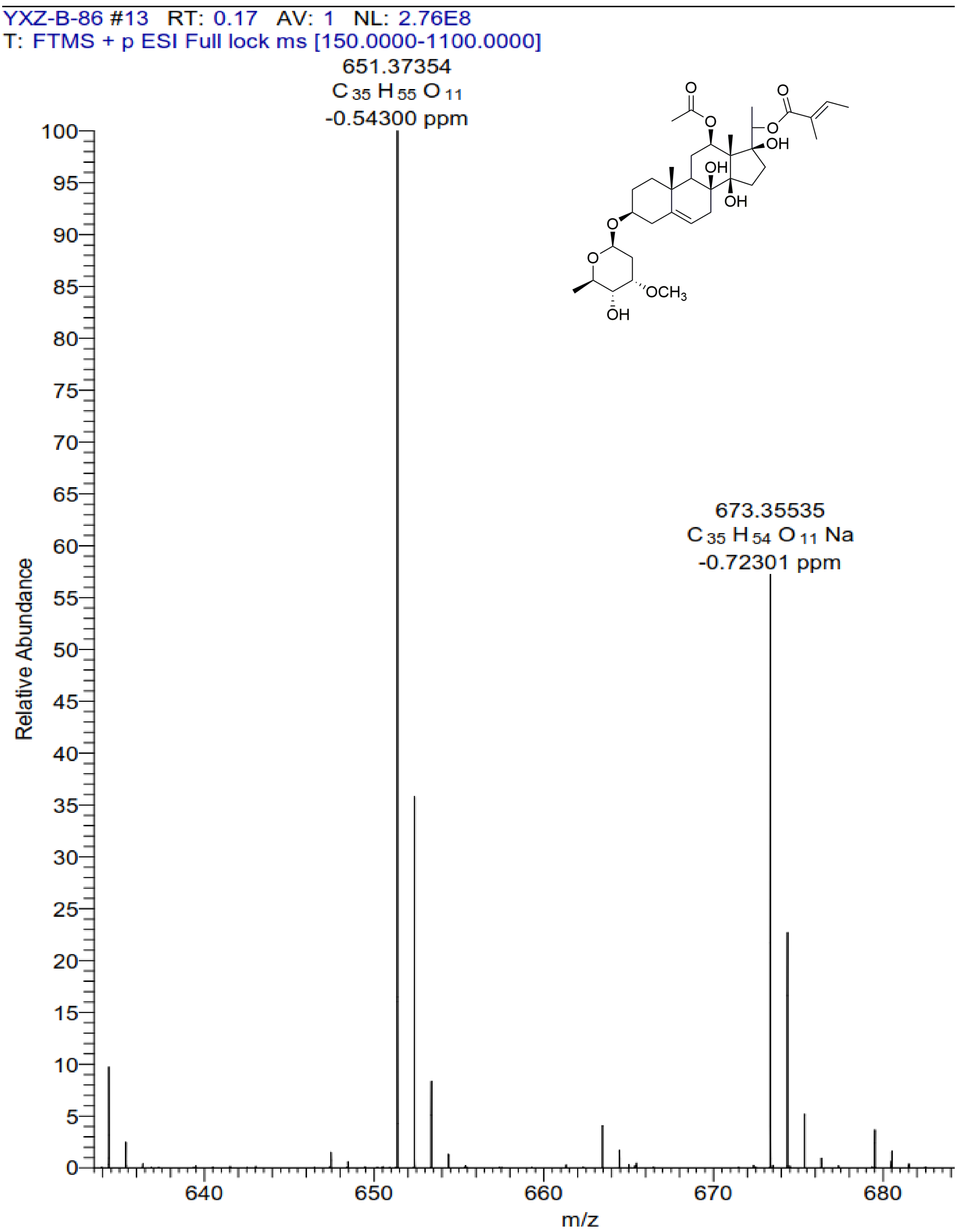

Figure S37 HRESIMS spectrum of **4**

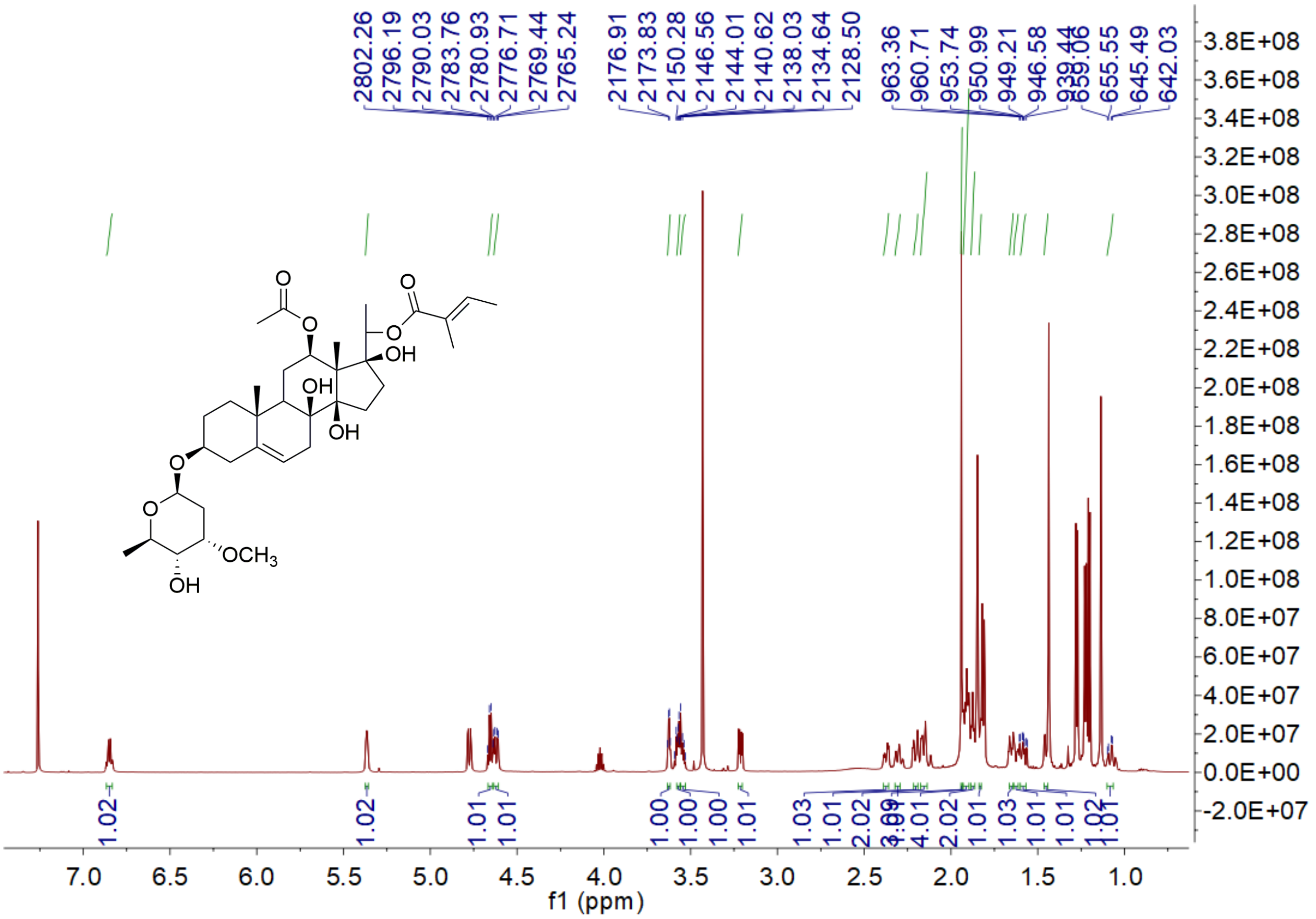

Figure S38 ^{1}H NMR spectrum (600 MHz, $CDCl_3$) of **4**

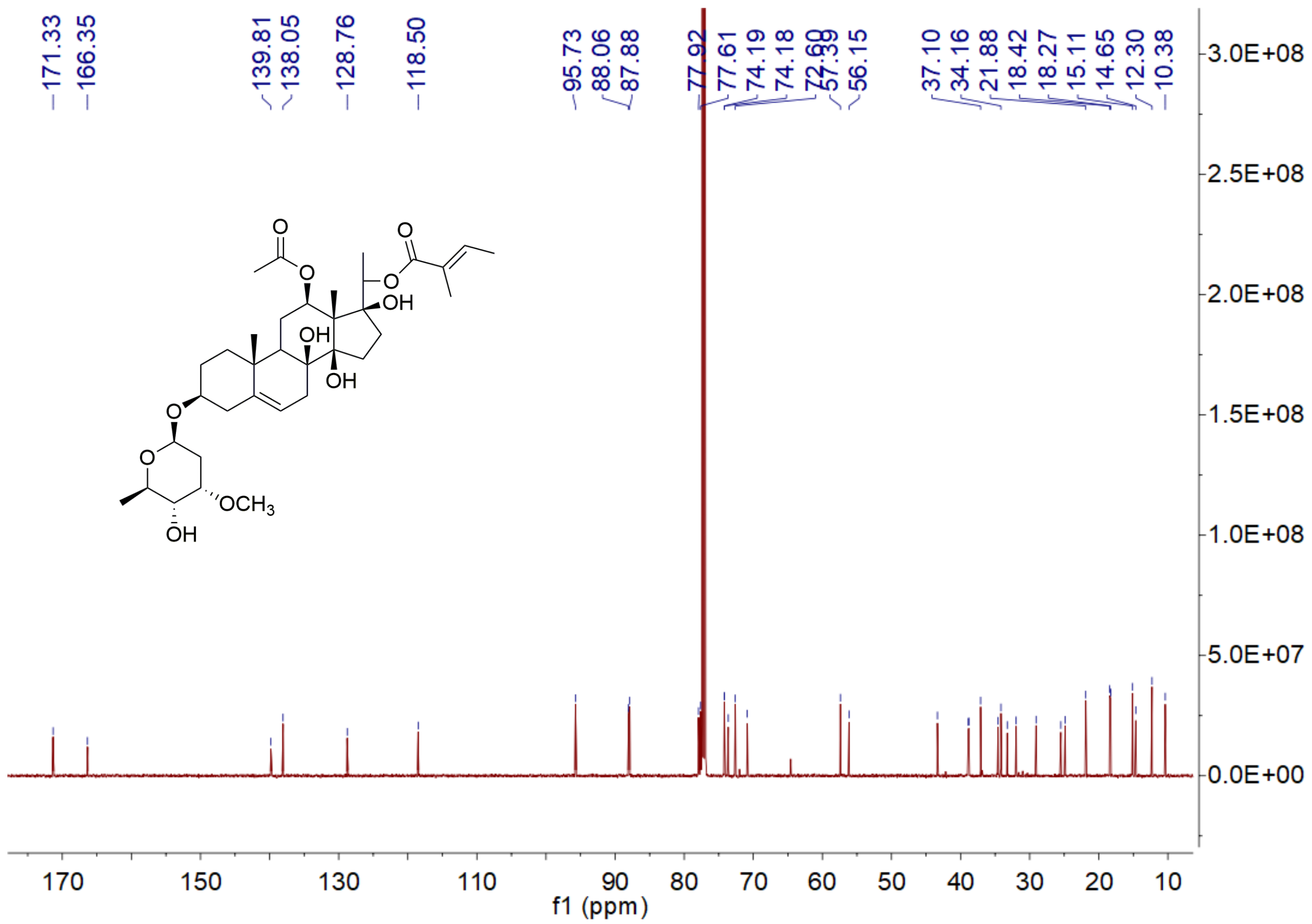

Fingue S39 ^{13}C NMR spectrum (150 MHz, $CDCl_3$) of **4**

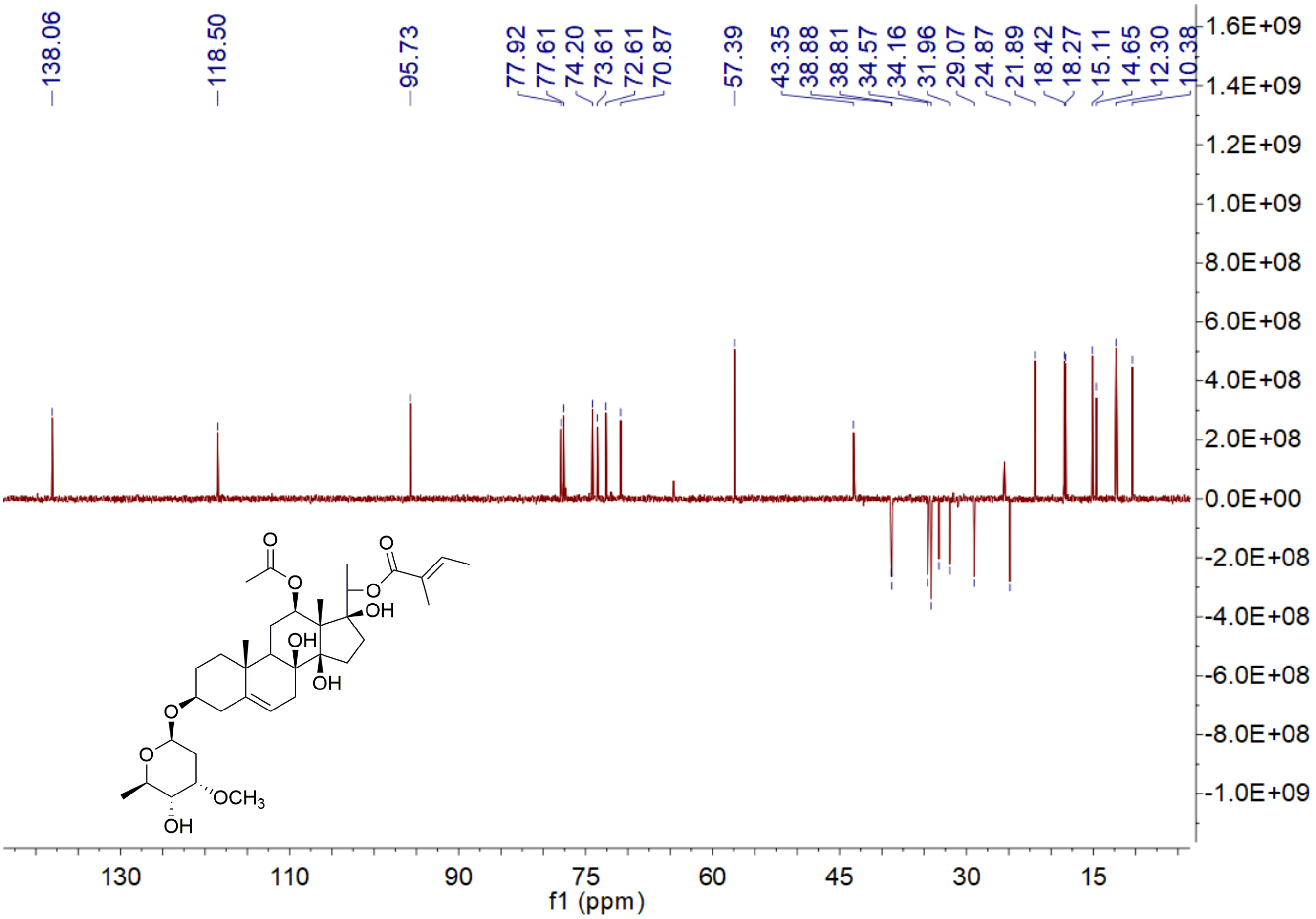

Figure S40 DEPT 135° spectrum (150 MHz, $CDCl_3$) of **4**

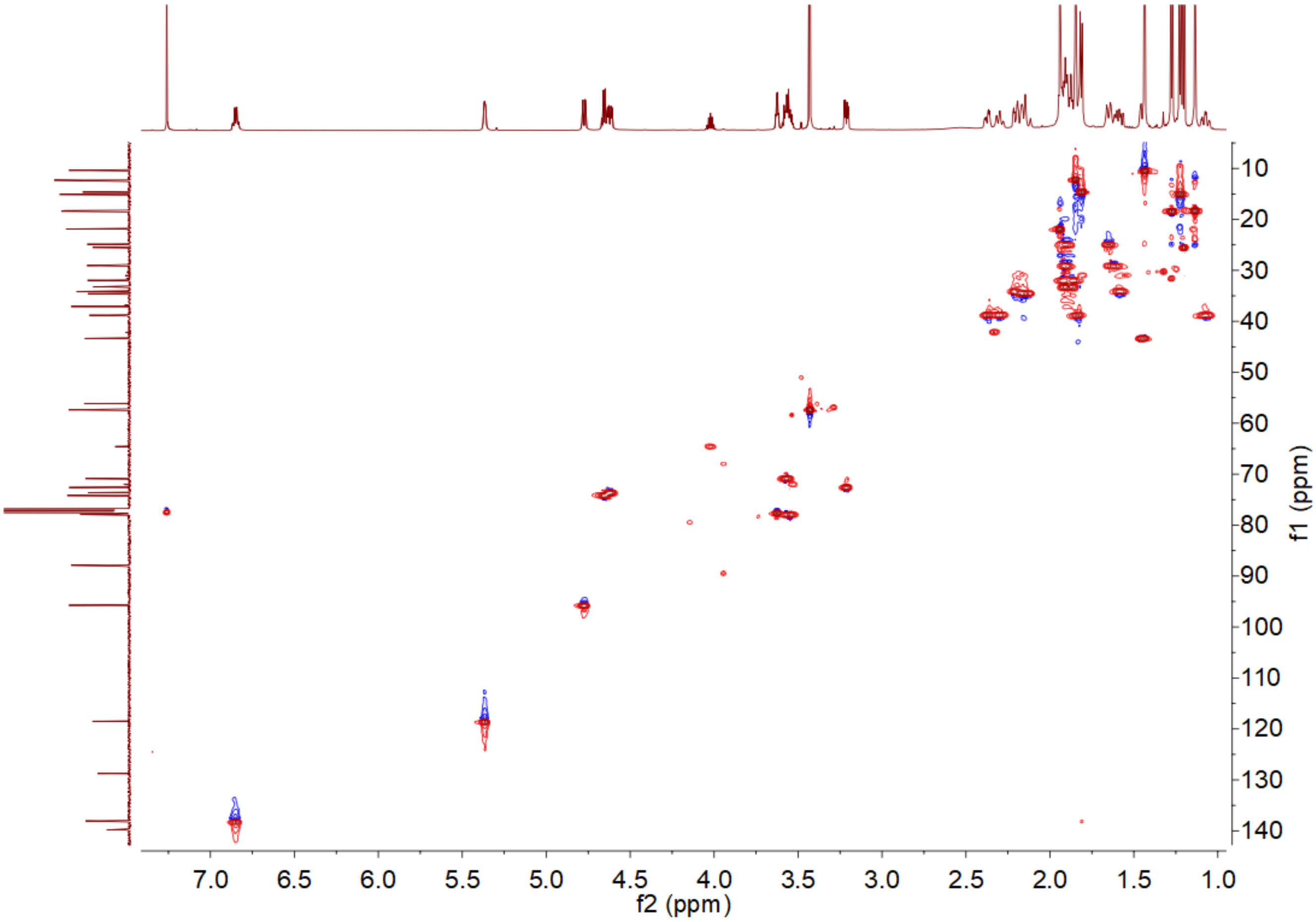

Figure S41 HSQC spectrum of **4**

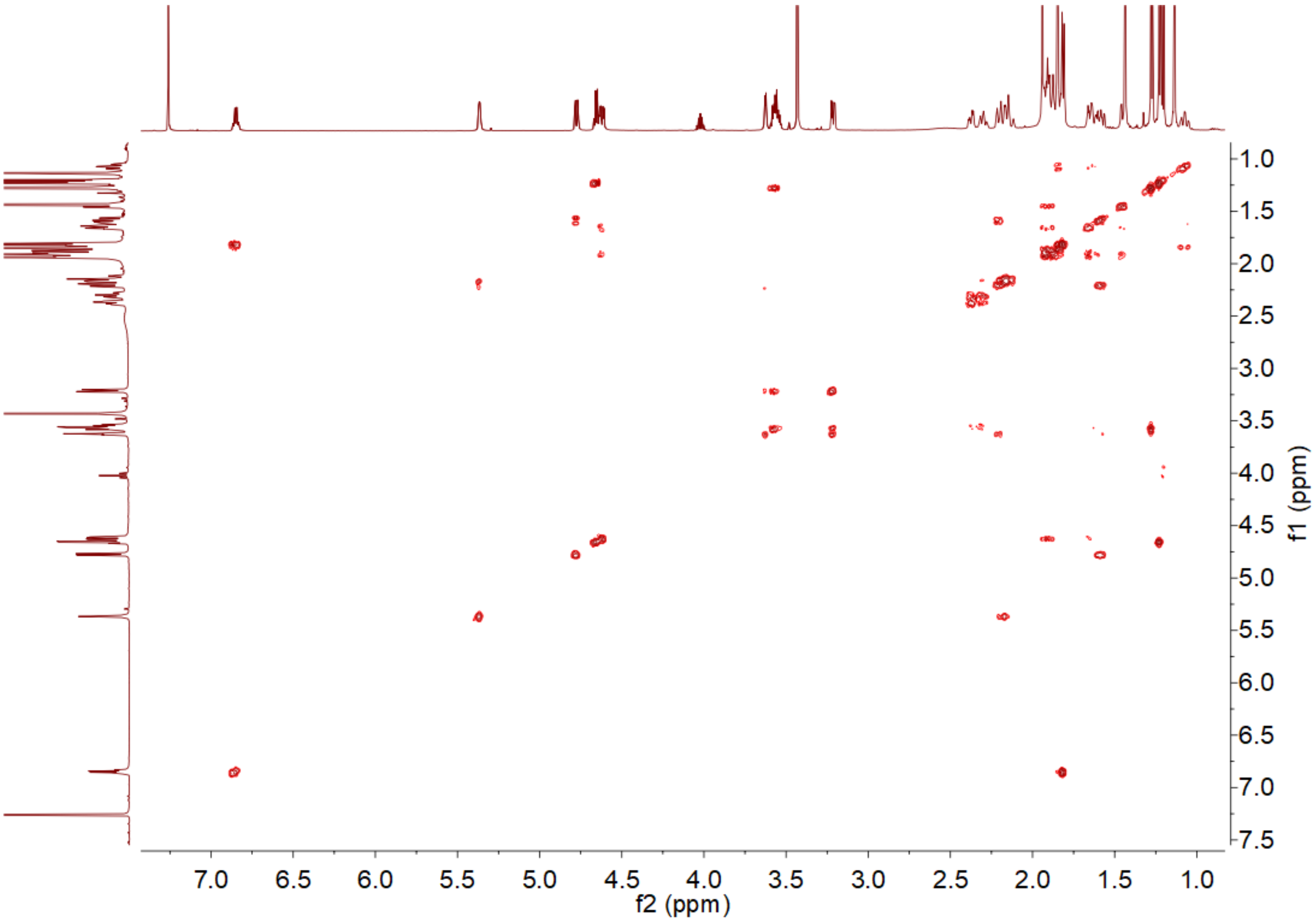

Figure S42 COSY spectrum of **4**

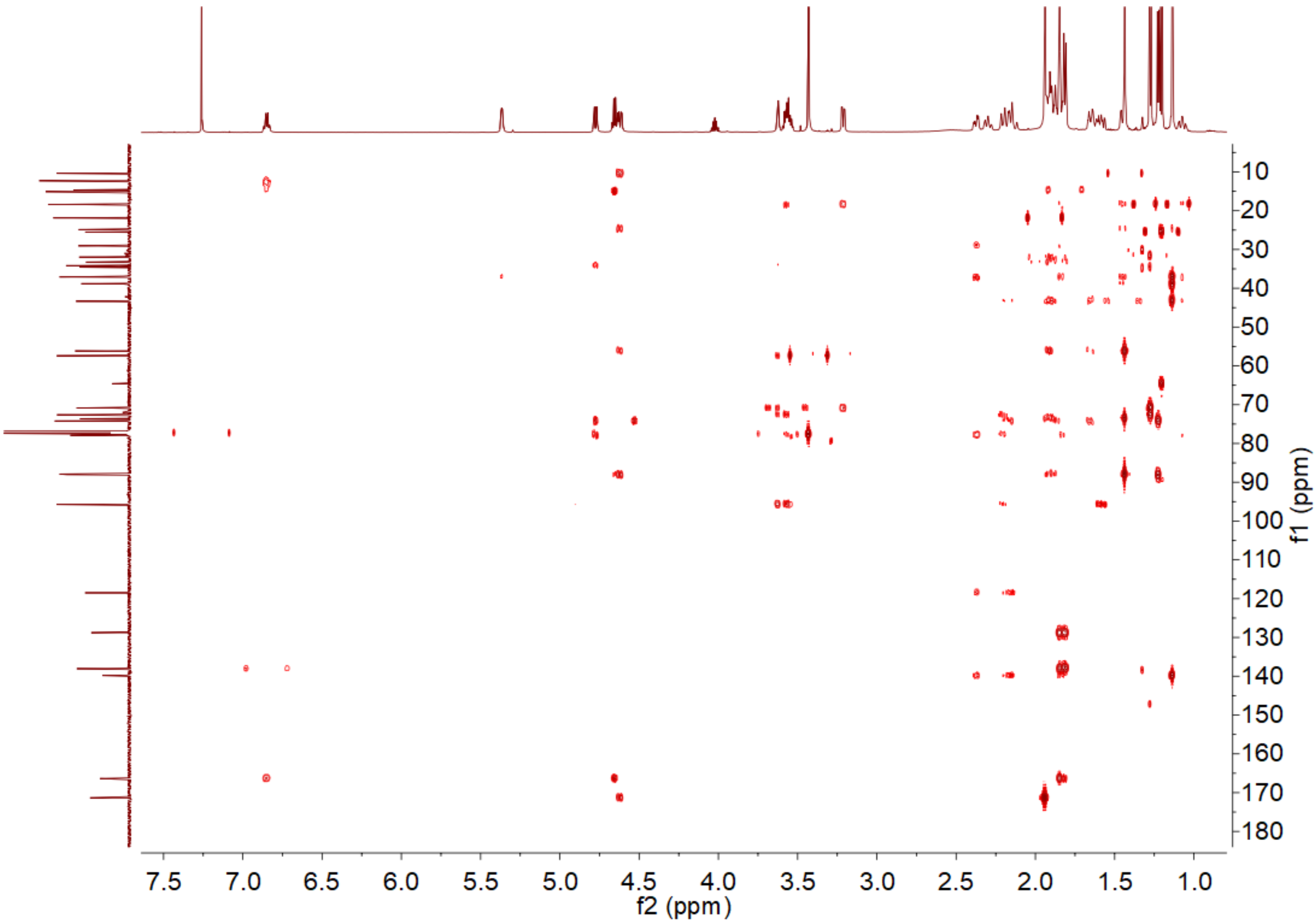

Figure S43 HMBC spectrum of **4**

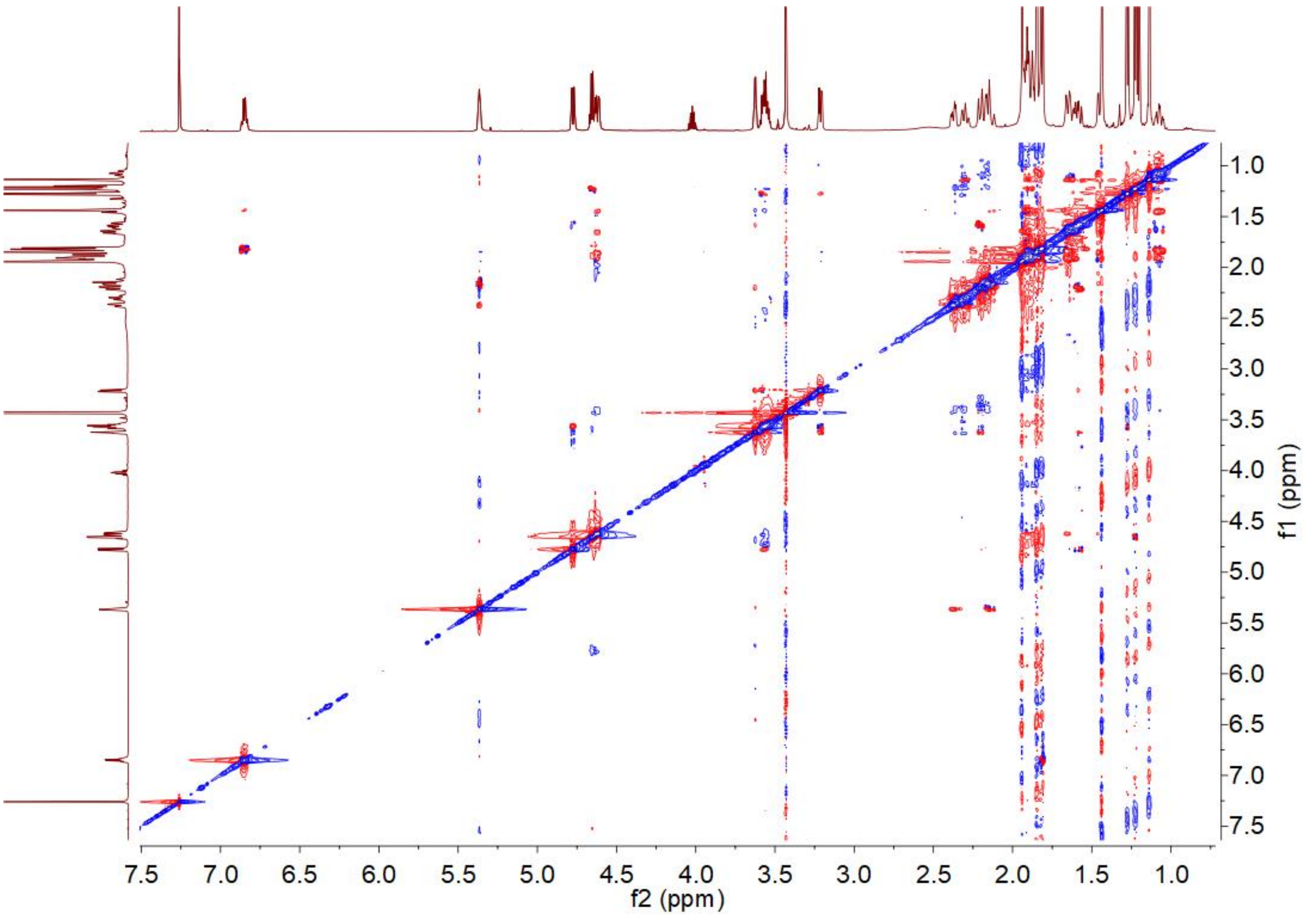

Figure S44 NOESY spectrum of **4**

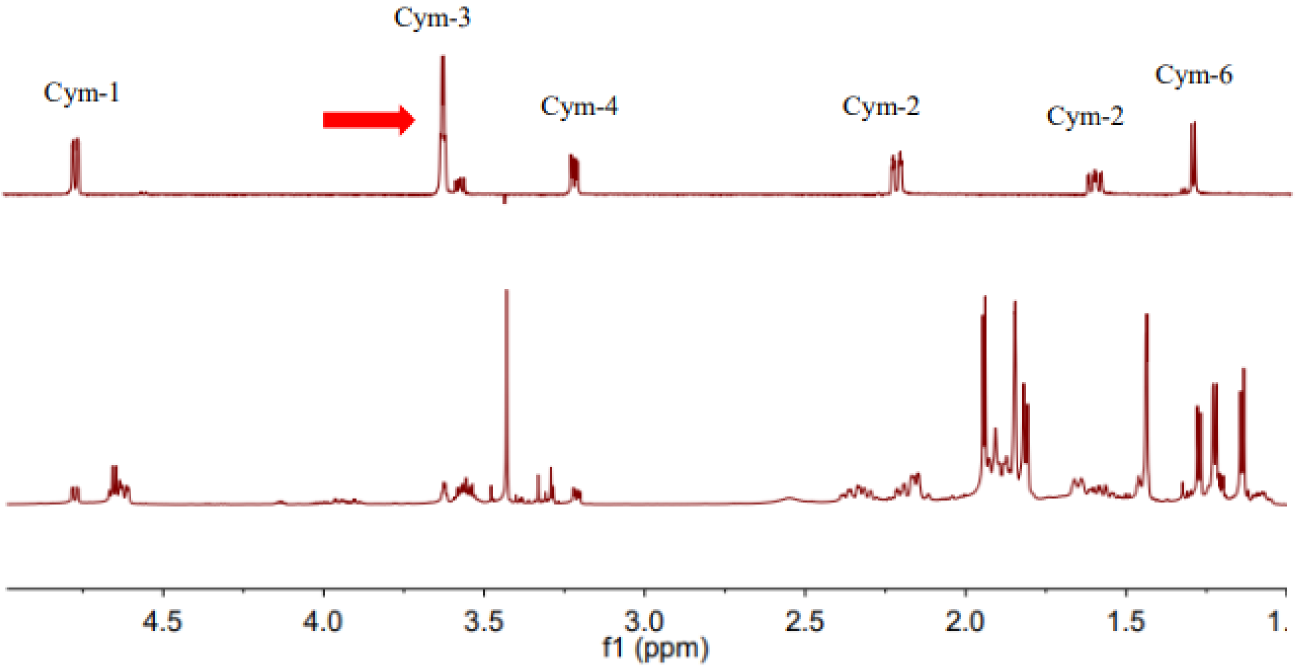

Figure S45 1D TOCSY spectrum of **4**

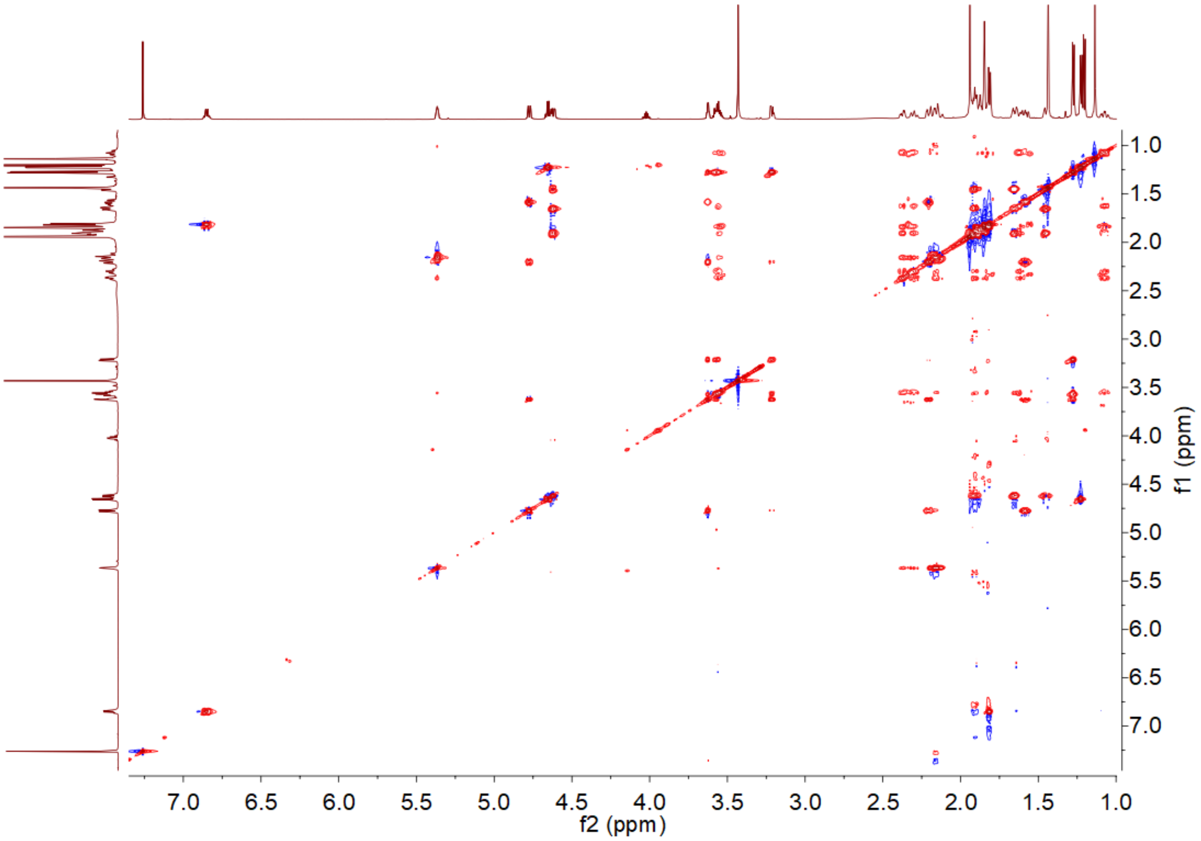

Figure S46 2D TOCSY spectrum of **4**

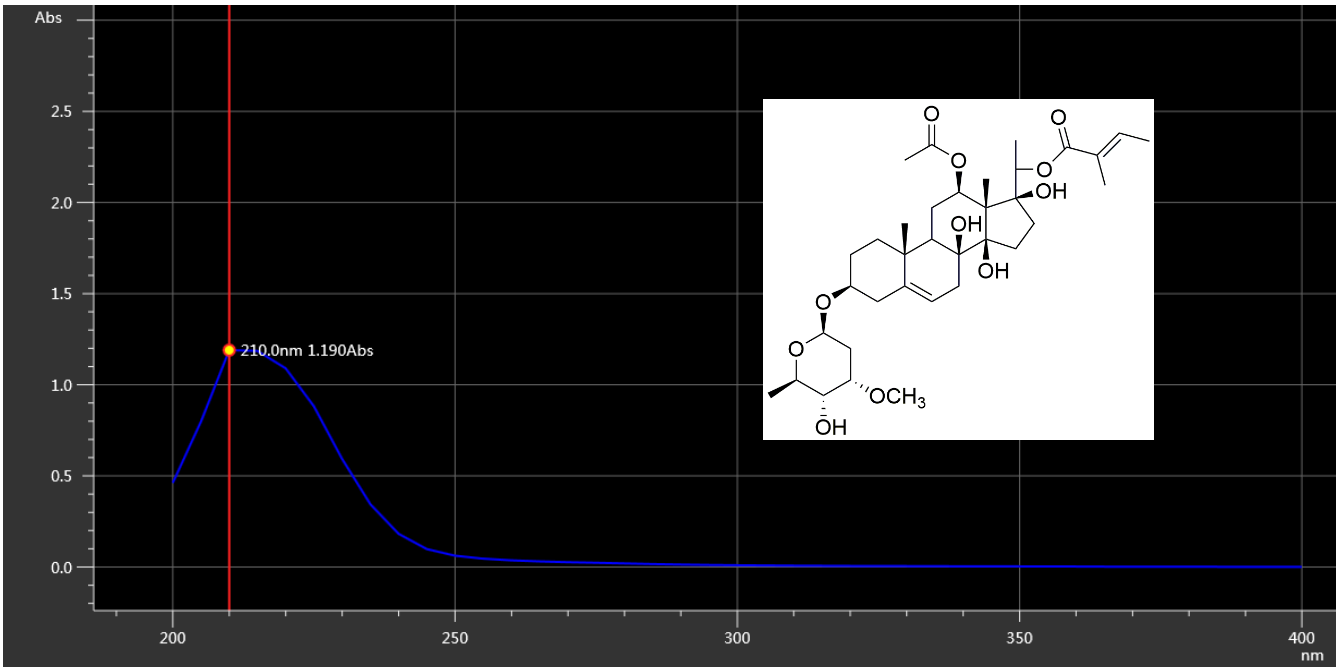

Figure S47 UV spectrum of **4**

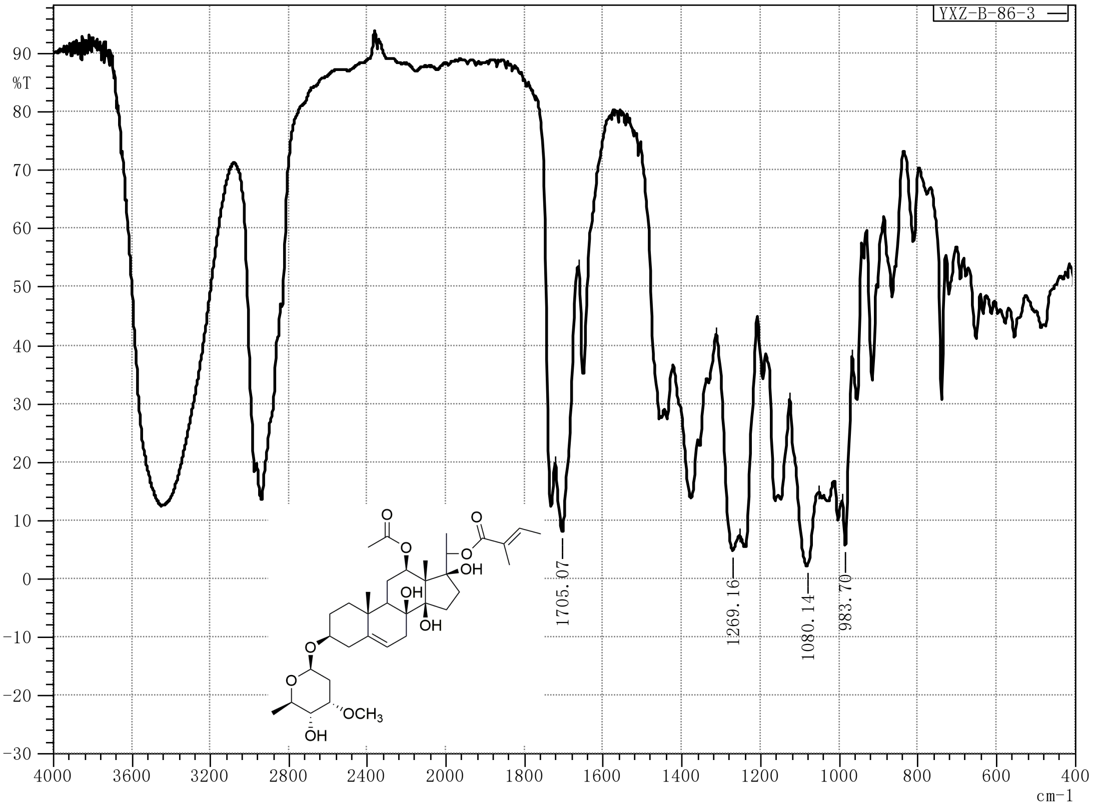

Figure S48 IR spectrum of **4**
